# A practical strategy to design and develop an isoform-specific fluorescent probe for a target enzyme: CYP1A1 as a case study†
†Electronic supplementary information (ESI) available: Detailed experimental procedures and characterization of compounds. See DOI: 10.1039/c6sc03970g



**DOI:** 10.1039/c6sc03970g

**Published:** 2016-12-19

**Authors:** Zi-Ru Dai, Lei Feng, Qiang Jin, Hailing Cheng, Yan Li, Jing Ning, Yang Yu, Guang-Bo Ge, Jing-Nan Cui, Ling Yang

**Affiliations:** a Dalian Institute of Chemical Physics , Chinese Academy of Sciences , Dalian , China . Email: yling@dicp.ac.cn ; Email: geguangbo@dicp.ac.cn; b Graduate School of Chinese Academy of Sciences , Beijing , China; c State Key Laboratory of Fine Chemicals , Dalian University of Technology , Dalian , China; d Cancer Institute , The Second Hospital of Dalian Medical University , Dalian , China

## Abstract

The development of isoform-specific probe(s) for a target enzyme with multiple homologs is always challenging. Herein, a practical strategy was used to design and develop an isoform-specific probe for CYP1A1, a key cytochrome P450 isoenzyme involved in xenobiotic metabolism and bioactivation. On the basis of the subtle differences in 3D structure and substrate preference between CYP1A1 and its homolog CYP1A2, we proposed that it was possible to design a CYP1A1-specific probe *via* local modification of the reaction site on known CYP1A substrates. To validate this hypothesis, 4-hydroxy-1,8-naphthalimide (**HN**) was selected as the basic fluorophore due to its excellent optical properties, while a series of *O*-alkylated **HN** derivatives were prepared to evaluate their specificity towards CYP1A1. Our results revealed that the introduction of a chloroethyl to **HN** could get the best isoform selectivity towards CYP1A1 over other CYPs including CYP1A2. The newly developed probe **NBCeN** exhibited excellent specificity, high sensitivity, and a ratiometric fluorescence response following CYP1A1-catalyzed *O*-dechloroethylation. **NBCeN** was successfully used to real-time monitor the activity of CYP1A1 in complex biological samples and to rapidly screen CYP1A1 modulators in living systems. **NBCeN** could also be used for two-photon imaging of intracellular CYP1A1 in living cells and tissues with high ratiometric imaging resolution and deep tissue penetration. All these findings demonstrated that local modification of non-specific substrates was a practical strategy to develop an isoform-specific probe for a target isoenzyme, while **NBCeN** could serve as a specific imaging tool to explore the biological functions of CYP1A1 in complex biological systems.

## Abstract 2

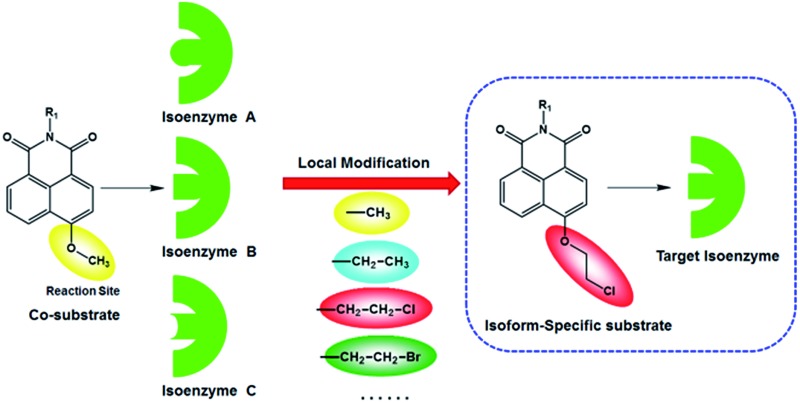
A practical strategy was proposed and successfully used to design and develop an isoform-specific probe for a given enzyme.

## Introduction

The human proteome projects have uncovered more than 20 000 proteins distributed in human tissues and cells.^1^ It is noteworthy that a large fraction (72%) of human genes encode multiple variants with similar but different protein sequences, which means that most human proteins have more than one isoform.^2^ As an important class of human proteins, enzymes catalyze more than 6000 reactions in the human body and play very important roles in both exogenous and endogenous metabolism, and thus are recognized as key factors affecting human physiological and pathological processes.^3^ It is well-known that deciphering the physiological functions of target enzyme(s) requires specific tools (such as probe substrates and selective inhibitors) to profile and perturb their activities in native biological systems.^4^ However, the members belonging to an enzyme subfamily always have similar structures and display very minor differences in function (such as reaction types and substrate specificity), thus it is very hard for biochemists to find isoform-specific probes for a target enzyme with multiple homologs.^5^ Although several methods including antibody-based assays and mass spectrometry-based proteomic techniques can be used to quantify the levels of many enzyme isoforms in various biological samples, these methods cannot be used to characterise the biological functions of a target enzyme.^6^ Although many activity-based probes including optical and non-optical substrates have been designed and used to directly measure the enzymatic activities of target enzyme(s) in various biological systems, almost all reported probes are not designed by targeting a specific isoform.^7^ Therefore, it is urgently necessary to develop a practical strategy to guide the rational design of isoform specific probes for a target enzyme.

The cytochrome P450 (CYP) superfamily is the largest enzyme family in mammals consisting of over 750 heme-containing membrane proteins located primarily in the endoplasmic reticulum (ER).^8^ In mammals, CYP monooxygenases catalyze the oxidative metabolism of endogenous compounds and various xenobiotics, leading to the metabolic activation and detoxification of a wide variety of environmental pollutants and drugs.^9^ The CYP1A subfamily plays critical roles in the metabolic activation of a great variety of procarcinogenic compounds to endotoxic intermediates or ultimate carcinogens, and thus has attracted increasing attention in the fields of toxicology and oncology.^10^ In humans, the CYP1A subfamily consists of two major isoforms including CYP1A1 and CYP1A2.^11^ CYP1A2 is abundantly expressed in the liver, while CYP1A1 is expressed in hepatic and extrahepatic tissues including the lung salivary gland and the gastrointestinal tract.^12^ Previous studies have clearly shown that CYP1A1 contributes to tumour formation and cancer susceptibilities.^13^ To better understand the biological and physiological roles of CYP1A1, it is necessary to develop isoform-specific probe(s) for highly selective and sensitive detection of the real activities of CYP1A1 in complex biological systems.

In the last decade, activity-based optical probes offer promising tools to monitor the real activities of target enzymes under physiological conditions, due to their high selectivity, non-destructiveness, as well as the capacity for high-throughput real-time monitoring.^7c,14^ Among various types of optical imaging techniques, two-photon microscopy (TPM), by virtue of its higher sensitivity, real-time spatial high-resolution imaging, and reduced phototoxicity or photodamage to biological samples, has shed new light on real-time monitoring and imaging of target enzyme activity.^15^ Therefore, it is highly desirable to take advantage of TPM imaging for precise detection of the real activities of target enzyme(s), especially in complex biological systems.^16^

CYP1A1 and CYP1A2 share high homology (72.55%) in amino acid sequence and show overlapped substrates and inhibitor spectra.^5^ To the best of our knowledge, isoform-specific probes for CYP1A1 have not been reported yet. In this study, we aimed to develop a CYP1A1-specific probe which can be used to explore the biological functions and physiological roles of this isoenzyme in living systems. From the 3D structures of two human CYP1A isoforms, CYP1A1 has a rather planar active site with a cavity volume of 524 Å, which is larger than that of CYP1A2 (375 Å).^17^ Furthermore, both CYP1A1 and CYP1A2 are typical dealkylating enzymes; CYP1A2 shows a certain predilection for demethylation, while CYP1A1 can catalyze *O*-dealkylation of relatively large groups, such as *O*-ethyl or *O*-chloroethyl.^18^ These findings indicate that the catalytic cavity of CYP1A1 is larger and more flexible than that of CYP1A2, while CYP1A1 can catalyze substrates with relatively large *O*-alkyl groups. These previous findings prompted us to develop an isoform-specific probe for CYP1A1 *via* adjusting the leaving groups (such as the *O*-alkyl group).

**Scheme 1:**
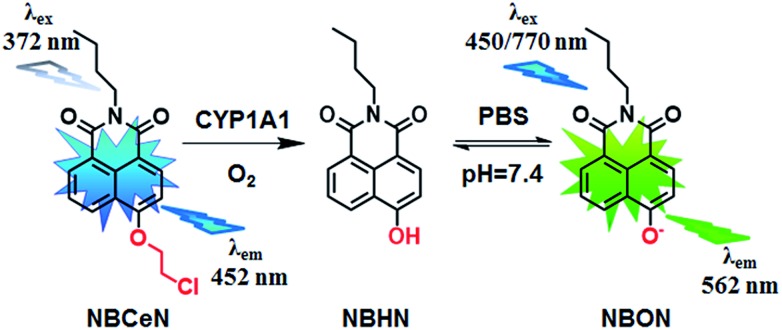
Structure of **NBCeN** and its fluorescence response towards CYP1A1.

Recently, we reported that 4-methoxy-1,8-naphthalimide and its derivatives are good substrates of CYP1A, but they could not distinguish between CYP1A1 and CYP1A2.^19^ Herein, to design and develop an isoform specific two-photon fluorescent probe for CYP1A1, a practical strategy was employed *via* adjusting the local structure of the reaction site on this known CYP1A fluorescent substrate. To this end, 4-hydroxy-1,8-naphthalimide (**HN**) was selected as the basic fluorophore, while a series of *O*-alkylated **HN** derivatives were designed and screened. Following both molecular docking-based virtual screening and reaction phenotyping-based experimental screening, *N*-(4-butyl)-4-chloroethoxy-1,8-naphthalimide (**NBCeN**) displayed good reactivity and high selectivity towards CYP1A1 over other CYP isoforms including CYP1A2. Under physiological conditions, the chloroethyl group of **NBCeN** could be readily cleaved by CYP1A1, leading to the release of *N*-(4-butyl)-4-hydroxy-1,8-naphthalimide (**NBHN**). As a result, CYP1A1-mediated **NBCeN**-*O*-dechloroethylation triggered remarkable changes in both colour and fluorescence emission (Scheme 1). Based on these findings, we further characterized the features of this isoform-specific probe for CYP1A1, and explored the feasibility and practicability of its use in complex biological systems.

## Results and discussion

### Design and synthesis of **NBCeN**

Given that the active cavity of CYP1A1 is larger than that of CYP1A2 and that CYP1A1 prefers dealkylation of larger *O*-alkyl groups, a library of *O*-alkylated **HN** derivatives were purposely designed and then evaluated for their potential as good substrates for CYP1A1 through molecular docking-based virtual screening and reaction phenotyping-based experimental screening.^20^ Representative structures and chemical characteristics are depicted in Fig. S1 (ESI†), including methyl (1), ethyl (2), propyl (3), isopropyl (4), butyl (5), chloroethyl (6), chloropropyl (7), bromoethyl (8), five-membered ring (9), six-membered ring (10), benzene ring (11), thiophene (12) and pyridine (13). The molecular docking results demonstrated that the alkyl groups of compounds (1)–(8) could orient towards the active sites of CYP1A1 and CYP1A2 (with catalytically reactive distances from the proton-iron of CYP450 ranging from 2.5 to 5.5 Å). In particular, compound (6) could orient itself markedly better in the active site of CYP1A1 than that of CYP1A2 (Tables S1 and S2, ESI†). After then, reaction phenotyping screening using a panel of human CYPs revealed that *O*-alkylated **HN** derivatives (1)–(8) displayed good reactivity towards CYP1A, but most of them could also be rapidly *O*-demethylated by other CYPs including CYP2B6, CYP2D6 and CYP3A4. Among these derivatives, *N*-(4-butyl)-4-chloroethoxy-1,8-naphthalimide (6, **NBCeN**) displayed good reactivity and high selectivity towards CYP1A1 over other CYPs isoforms, especially CYP1A2 (Table S3, ESI†). The chloroethoxy group of **NBCeN** could be selectively cleaved by CYP1A1, leading to the release of *N*-(4-butyl)-4-hydroxy-1,8-naphthalimide (**NBHN**). The chemical structure and ratiometric fluorescence mechanism of the probe **NBCeN** was depicted in Scheme 1. **NBCeN** could be readily synthesized using **NBHN** and 1-chloro-2-iodoethane as the starting materials (Scheme S1, ESI†), while its structure was fully characterized by ^1^H-NMR, ^13^C-NMR and HRMS (ESI†).

### Spectral properties of **NBCeN** towards CYP1A1

**Fig. 1:**
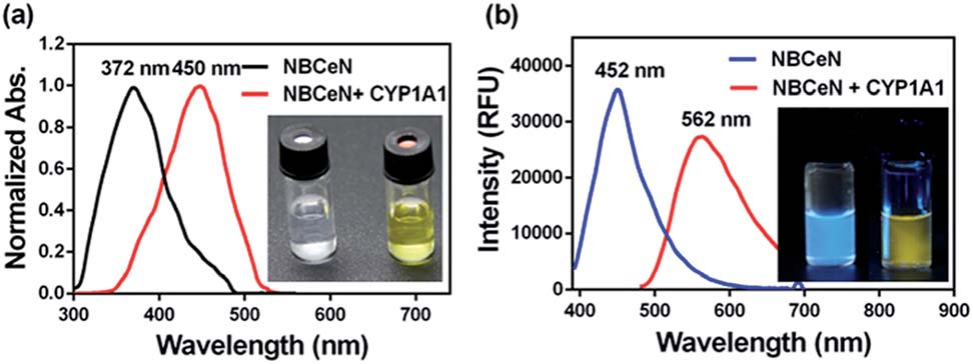
(a) Normalized absorption spectra of **NBCeN** (20 μM) upon addition of CYP1A1 (100 nM). (b) Emission spectra of **NBCeN** (20 μM) upon addition of CYP1A1 (100 nM).

**NBCeN** was found readily dealkylated upon addition of CYP1A1, or CYP1A1-containing tissue preparations including human liver microsomes (HLM) and lung microsomes (HLuM). Meanwhile, a single metabolite **NBHN** was identified by comparison of LC retention times, UV and MS spectra with the help of a standard (Fig. S2, ESI†). Notably, the formation of **NBHN** was time-, NADPH-, and enzyme-dependent. As shown in Fig. 1a and b, **NBCeN**-*O*-dechloroethylation brought a remarkable fluorescence enhancement at 562 nm (for **NBHN**), accompanied by a gradual decrease in the emission peak at 452 nm (for **NBCeN**). The large red shift (110 nm) in the emission behaviour led to a striking change of the reaction from colourless to yellow, indicating that **NBCeN** could serve as a ‘‘naked-eye’’ colorimetric indicator for target enzyme(s). Furthermore, the effects of organic solvent and pH on the fluorescence response of **NBCeN** and **NBHN** were also studied. DMSO at low concentration (0.5%) did not have any obvious effects on the formation rate of **NBHN** (Fig. S3, ESI†). In addition, while **NBHN** was quite stable over the pH range of 7.0–10.0, **NBCeN** was stable in a much wider pH range between 2.0 and 10.0 (Fig. S4, ESI†). Together, these results demonstrated that **NBCeN** could function properly under physiological conditions (pH 7.4) at 37 °C.

### Isoform specificity of **NBCeN** towards CYP1A1

**Fig. 2:**
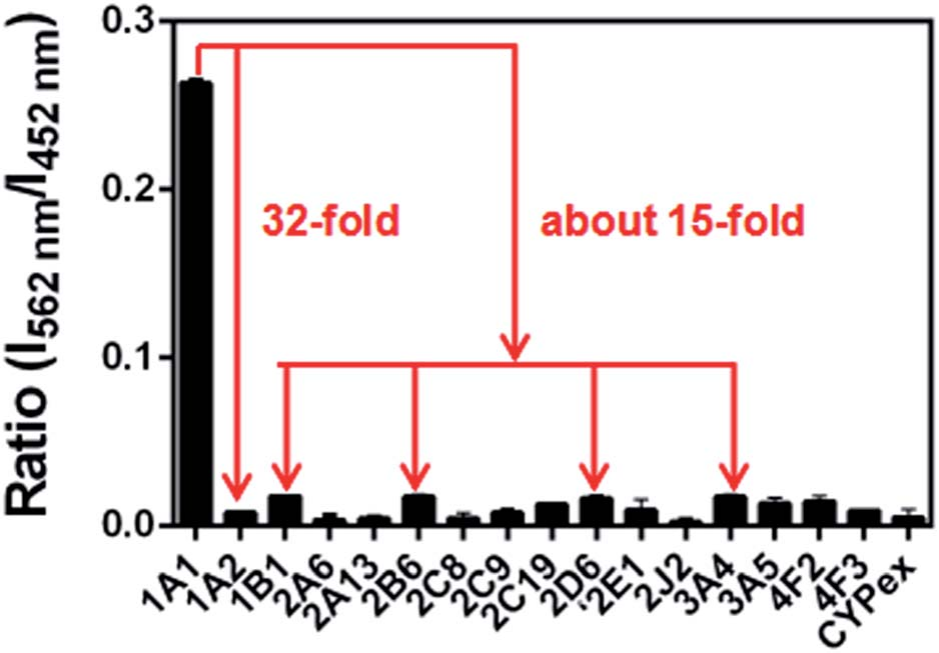
Fluorescence responses of **NBCeN** (20 μM) towards various species of enzymes in human tissue microsomes.

The selectivity of **NBCeN** towards CYP1A1 was then examined under physiological conditions (pH 7.4 at 37 °C). A series of CYP isoforms were used to evaluate their possible participation in the formation of **NBHN**. As shown in Fig. 2, among all CYP isoforms examined, only CYP1A1 triggered a remarkable change in the fluorescence spectrum. Indeed, there was 32-fold and 15-fold difference in fluorescence intensity for CYP1A1 compared to CYP1A2 and other CYPs (*e.g.* 1B1, 2B6, 2D6 and 3A4), respectively. Moreover, the fluorescence response of **NBCeN** to various biologically relevant small molecules was also evaluated to further explore its anti-interference ability in complex biological systems. As shown in Fig. S5 (ESI†), the excellent responsiveness of **NBCeN** towards CYP1A1 was not affected in the presence of common biological metallic ions or amino acids in human tissues or fluids. These results revealed that **NBCeN** was highly selective for CYP1A1 over other biologically relevant species. To further validate the selectivity of **NBCeN** in complex biological systems, chemical inhibition assays were conducted in human tissue preparations using selective inhibitors of major human CYP isoforms. As shown in Fig. S6 (ESI†), the formation of **NBHN** could be potently inhibited by ABT (a broad CYP inhibitor) and resveratrol (a selective inhibitor of CYP1A1), while furafylline (a potent inhibitor of CYP1A) exhibited lower inhibitory effects on **NBCeN**-*O*-dechloroethylation than resveratrol.^21^ In contrast, inhibitors of other CYP isoforms exhibited minor inhibitory effects on this (CYP1A1 mediated) biotransformation. All these results supported **NBCeN** as a highly selective substrate for human CYP1A1.

### Fluorescence sensing behaviour of **NBCeN** towards CYP1A1

**Fig. 3:**
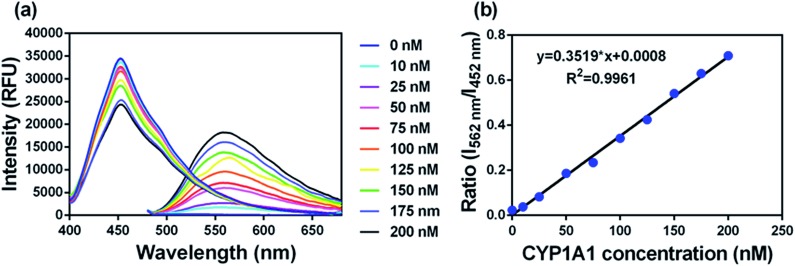
(a) The changes in fluorescence spectra and (b) fluorescence intensity ratios (*I*_562_/*I*_452_) of **NBCeN** (20 μM) upon addition of increasing concentrations of CYP1A1 (0–200 nM) in PBS–acetonitrile (v/v = 1 : 1, pH 7.4) at 37 °C. *λ*_ex_ = 372/452 nm.

To accurately measure the enzyme activity, the linear response ranges for CYP1A1 quantification with **NBCeN** as a probe substrate were evaluated under physiological conditions. Time course studies showed that the formation rates of **NBHN** in CYP1A1 were linearly related to the reaction time, and the ratio of fluorescence intensities (562 nm/452 nm) showed good linearity (*R* ^2^ > 0.99) with the incubation time up to 30 min (Fig. S7, ESI†). Therefore, further assays related to quantitative analysis for CYP1A1 were conducted within 30 min. The responses of **NBCeN** towards target enzymes with different concentrations were also determined. An increased CYP1A1 concentration resulted in a remarkable enhancement in fluorescence at 562 nm, while the original emission peak of **NBCeN** at 452 nm decreased gradually. As shown in Fig. 3, a good linear relationship (*R* ^2^ > 0.99) between the fluorescence intensity ratio (562 nm/452 nm) and enzyme concentrations in the range of 0 to 200 nM were presented. The corresponding detection limit of **NBCeN** for CYP1A1 was evaluated as 2.5 nM (Fig. S8, ESI†). The excellent selectivity and sensitivity of the fluorescence-based assay implied that **NBCeN** could serve as an efficient tool for the specific detection of CYP1A1 enzyme activity in biological samples.

**Fig. 4:**
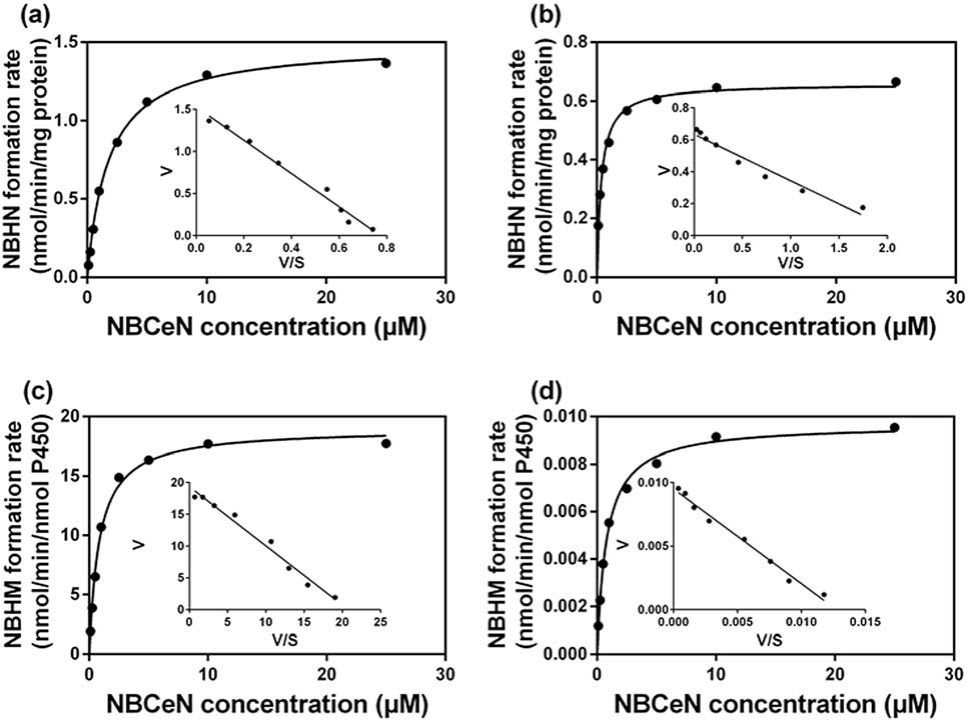
Michaelis–Menten plots of **NBCeN**-*O*-dechloroethylation in human liver microsomes (a) and human lung microsomes (b), CYP1A1 (c) and CYP1A2 (d). The corresponding Eadie–Hofstee plot is shown as an inset.

It is well-known that kinetic behavior is very crucial for the quantitative applications of activity-based probe substrates.^22^ In this study, fluorescence intensity was used to characterize the enzymatic kinetics of **NBCeN**-*O*-dechloroethylation in different enzyme sources including HLM, HLuM, CYP1A1 and CYP1A2. **NBCeN**-*O*-dechloroethylation in these enzyme sources displayed typical Michaelis–Menten kinetics, as evidenced by the corresponding Eadie–Hofstee plots (Fig. 4). As shown in Table 1, **NBCeN**-*O*-dechloroethylation in both recombinant CYP1A and human tissue preparations displayed very high affinity (*K*_m_ < 2.0 μM). However, in contrast to CYP1A2, CYP1A1 showed good reactivity in **NBCeN**-*O*-dechloroethylation. Indeed, there was an estimated more than 1000-fold difference in catalytic efficacy and inherent clearance (*K*_cat_ */K*_m_) of **NBCeN**-*O*-dechloroethylation between CYP1A1 and CYP1A2. These results suggested that **NBCeN** was a highly specific probe for CYP1A1. Furthermore, HLuM displayed much lower reactivity for **NBCeN**-*O*-dechloroethylation than HLM, which could be attributed to the low protein levels of CYP1A1 in human lung tissues. Together, **NBCeN** appears to be a good substrate for CYP1A1 but not CYP1A2. The excellent selectivity and high affinity of **NBCeN** for CYP1A1 inspired us to use this probe for evaluation of CYP1A1 activity in biological samples containing multiple enzymes.

**Table 1:** Kinetic parameters of **NBCeN**-*O*-dechloroethylation determined in different enzyme sources^*a*^

Enzyme sources	*K*_cat_	*K*_m_	*K*_cat_/*K*_m_
HLM	1.49	1.79	837.07
HLuM	0.66	0.36	1805.56
CYP1A1	18.99	0.84	22 607.14
CYP1A2	0.01	0.81	20.12

^*a*^ *K*_cat_ values were in nmol min^–1^ mg^–1^ protein for liver and lung microsomes, or in nmol min^–1^ nmol^–1^ CYP for CYP1A1 and CYP1A2. The range of substrate concentrations was 0.1 to 25 μM. Each value was the mean ± S.D. of determinations performed in duplicate.

### Quantification of CYP1A1 in human tissue preparations

**Fig. 5:**
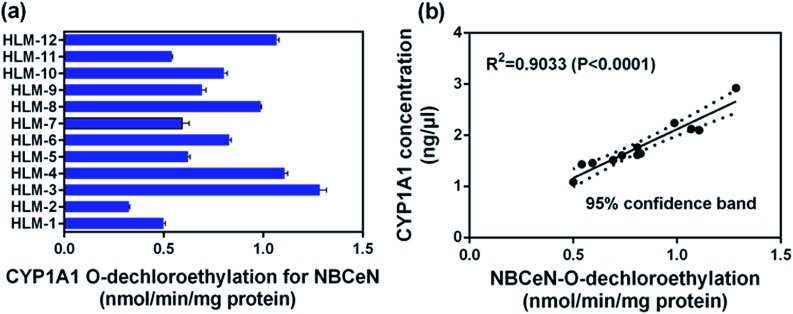
(a) The catalytic activities of **NBCeN** in a panel of twelve individual HLMs. (b) Correlation analysis between **NBCeN** (10 μM) *O*-dechloroethylation and the level of CYP1A1 in a panel of individual HLM samples (*n* = 12). The spectra were measured in PBS–acetonitrile (v/v = 1 : 1, pH 7.4).

To further evaluate the application of **NBCeN** as a probe, we next assessed the catalytic activities of CYP1A1 in individual HLM samples. The catalytic activities of CYP1A1 mediated **NBCeN**-*O*-dechloroethylation in a panel of twelve HLMs from different individuals were measured (Fig. 5a). Compared with the large differences in expression of CYP1A2, about 4-fold individual differences in CYP1A1 catalytic activity were observed, which agreed well with previously reported interindividual variability in CYP1A1 activity.^23^ In order to determine whether the formation rate of **NBHN** reflected the catalytic activities of CYP1A1 in these individual samples, we conducted a correlation analysis between the catalytic activities and the protein levels of CYP1A1 determined by a proteomics-based approach. As shown in Fig. 5b, a strong correlation with a high correlation parameter (*R* ^2^ = 0.9033, *P* < 0.0001) was presented between the *O*-dechloroethylation rates of **NBCeN** and the protein levels of CYP1A1 in a panel of 12 HLM samples. These findings strongly suggested that **NBCeN** could be used to measure the enzyme activity of CYP1A1 in complex biological systems.

### Rapid screening of CYP1A1 modulators

**Fig. 6:**
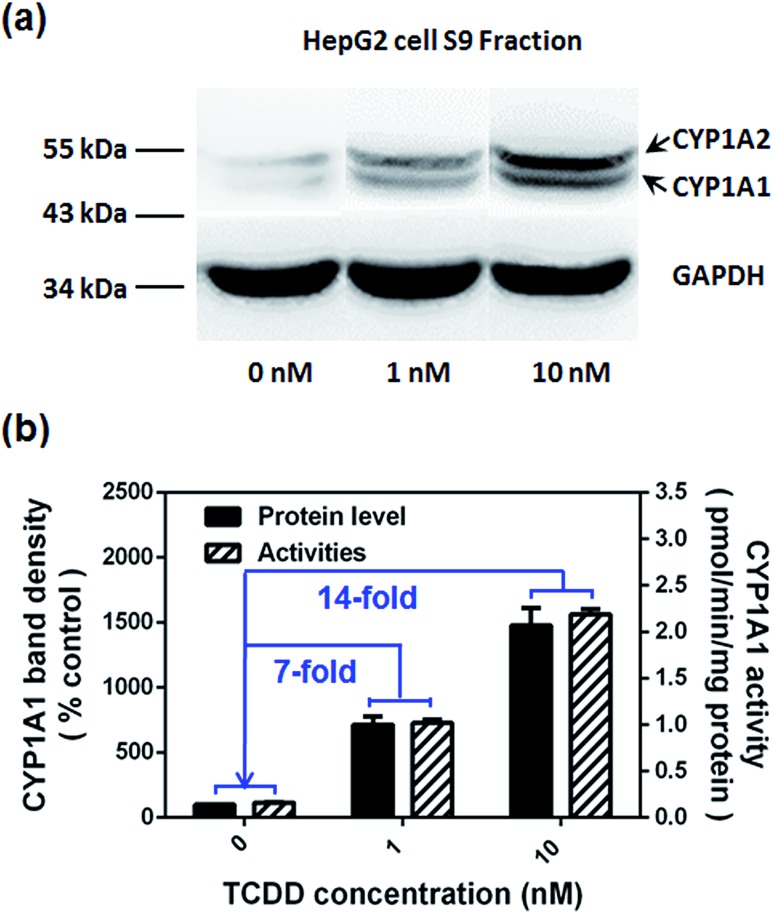
(a) The levels of the CYP1A1 protein in cell homogenates prepared from HepG2 pre-treated with TCDD (0, 1, 10 nM) were analyzed by western blot analysis. Data are shown as the mean ± S.D. (*n* = 3); (b) comparison of the catalytic activities and levels of CYP1A1 in HepG2 cells pre-treated with TCDD (0, 1, 10 nM).

Encouraged by the above mentioned findings, we explored the potential use of **NBCeN** as a fluorescent substrate for the screening of CYP1A1 inhibitors. One known chemical inhibitor of CYP1A1 (α-naphthoflavone) was used to evaluate its inhibitory effects toward CYP1A1 in both HLM and recombinant CYP1A1. As expected, this inhibitor inhibited CYP1A1-mediated **NBCeN**-*O*-dechloroethylation in a dose-dependent manner with similar inhibitory tendencies and IC_50_ values in these different enzyme sources (Fig. S11, ESI†), indicating that HLM could be used as an enzyme source instead of the more expensive recombinant CYP1A1 in such assays. In addition, **NBCeN** could also be used to characterise CYP1A1 inducers using fluorescence based high-throughput screening (HTS) assays. To explore this potential use, a known CYP1A1 inducer (2,3,7,8-tetrachlorodibenzo-*p*-dioxin, TCDD) was used to evaluate its effects on the expression and activity of CYP1A1 in HepG2 cells.^24^ As shown in Fig. 6, three different concentrations of TCDD (0, 1 and 10 nM) were used to stimulate the expression of CYP1A1. After co-incubation with TCDD for three days, there was a 14-fold increase in CYP1A1 activity in the cell homogenate upon addition of 10 nM TCDD (Fig. 6b). Furthermore, the elevated CYP1A1 activities were consistent with the increased protein levels of CYP1A1 determined by western blot analysis (Fig. 6a and b). These findings suggested that **NBCeN** could be used for high-throughput screening of potential CYP1A1 modulators using complex tissue or cell preparations as enzyme sources.

### Bioimaging of CYP1A1 in living cells

**Fig. 7:**
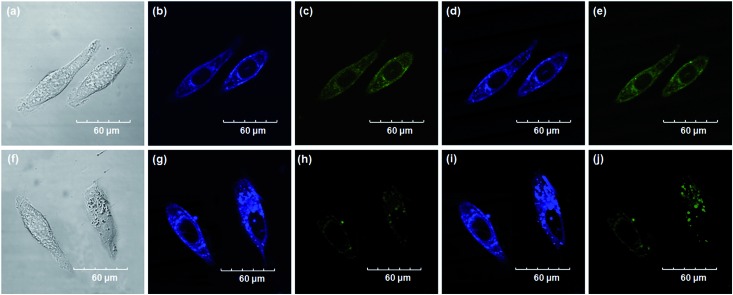
Confocal fluorescence images of A549 cells. Cells incubated with **NBCeN** (50 μM) for 1 h (top); cells pre-treated with resveratrol (50 μM) for 1 h and then incubated with **NBCeN** for 1 h (bottom). Single photon images were acquired using 405 nm excitation and fluorescent emission windows: (a) and (f) bright-field images; (b) and (g) blue emission channel; (c) and (h) green emission channel. Two photon images were acquired using 770 nm excitation and fluorescent emission windows: (d) and (i) blue = 420–460 nm; (e) and (j) green = 495–540 nm. Scale bar: 60 μm.

**Fig. 8:**
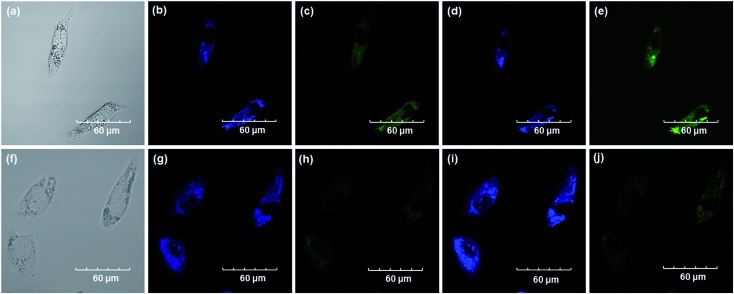
Confocal fluorescence images of SKOV3 cells. Cells incubated with **NBCeN** (50 μM) for 1 h (top); cells pre-treated with resveratrol (50 μM) for 1 h and then incubated with **NBCeN** for 1 h (bottom). Single photon images were acquired using 405 nm excitation and fluorescent emission windows: (a) and (f) bright-field images; (b) and (g) blue emission channel; (c) and (h) green emission channel. Two photon images were acquired using 770 nm excitation and fluorescent emission windows: (d) and (i) blue = 420–460 nm; (e) and (j) green = 495–540 nm. Scale bar: 60 μm.

Encouraged by the above mentioned findings, we next examined **NBCeN** for its application in bioimaging of endogenous CYP1A1 in living A549 and SKOV3 cells.^25^ Prior to the bioimaging of endogenous CYP1A1 in living cells, MTT assays were performed to evaluate the cytotoxicity of **NBCeN** towards A549 and SKOV3 cells. The results indicated that **NBCeN** exhibited relatively low toxicity towards the human cells examined, and the cell viabilities were about 80% upon addition of **NBCeN** (50 μM) at 37 °C for 48 h (Fig. S13, ESI†). We also investigated the induction effects of **NBCeN** on endogenous CYP1A1 in living cells. As shown in Fig. S14,† although the mRNA levels of CYP1A1 in A549 cells could be induced by **NBCeN** following 2 h incubation, the protein levels of CYP1A1 did not change following 2 h incubation. In these cases, **NBCeN** (50 μM) was co-incubated with A549 and SKOV3 cells and the confocal fluorescence images were recorded both in one and two-photon modes within 2 h. As shown in Fig. 7 and 8, after loading with **NBCeN** (50 μM) for 1 h at 37 °C, A549 and SKOV3 cells exhibited both single-photon and two-photon excited intense intracellular fluorescence in the blue and green channel (Fig. 7a–e and 8a–e), corresponding to fluorescence of the substrate at 452 nm and fluorescence of the product at 562 nm. In sharp contrast, upon pre-treatment of cells with the CYP1A1 selective inhibitor resveratrol (50 μM), a significant fluorescence decrease in green emission and a concomitant enhancement in the blue-channel were observed (Fig. 7f–j and 8f–j). It is evident that the changes in fluorescence response depend on the degree of intracellular CYP1A1-mediated **NBCeN**-*O*-dechloroethylation in various cell types. These findings suggested that **NBCeN** was cell membrane permeable and has low cell toxicity, and could be applied to both single-photon and two-photon-excited bio-imaging and for sensing the real-time activity of CYP1A1 in living cells.

### 3D depth imaging of CYP1A1 in rat liver tissues

**Fig. 9:**
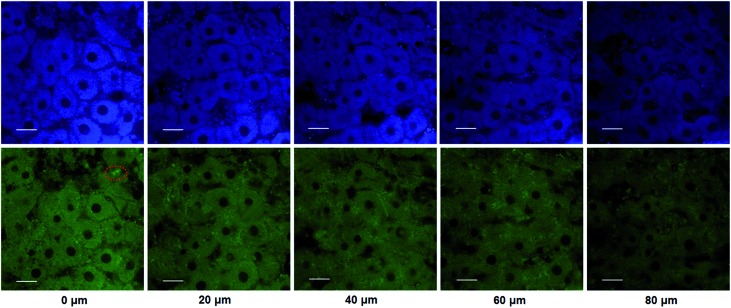
TPM images of a fresh rat liver slice stained with **NBCeN** (20 μM). Images were taken at a depth of 0–80 μm with magnification at 60×. Two photon images were acquired using 770 nm excitation and fluorescent emission windows: blue = 420–460 nm (top); green = 495–540 nm (bottom). Scale bar: 30 μm. Several little and anomalous bright fluorescence spots in the green channels have been marked, which may be ascribed to the endogenous matrix in rat liver.

The utility of this probe in tissue imaging was further investigated. Prior to tissue imaging, the specificity of **NBCeN** towards CYP1A1 in rat liver was evaluated. As shown in Fig. S16 (ESI†), **NBCeN**-*O*-dechloroethylation was significantly inhibited by known CYP1A1 inhibitors in rat liver preparation, validating CYP1A1 in rat liver as the key enzyme responsible for **NBCeN** dealkylation. The liver was isolated from a 7 week-old rat, and liver slices were incubated with 50 μM **NBCeN** for 1 h at 37 °C. As shown in Fig. 9, these TPM images revealed strong fluorescence responses in both blue and green channels at a depth of 80 μm, indicating that CYP1A1 was abundant in the rat liver. Several little and anomalous bright fluorescence signals in green channels have also been observed in TPM images of a fresh rat liver slice stained with or without **NBCeN** (Fig. 9 and S17†), which may be attributed to the endogenous matrix molecules in rat liver tissue that can be excited under the detection conditions. All these results indicated that **NBCeN** displayed good tissue penetrability and was appropriate for direct two-photon fluorescent depth imaging of CYP1A1 in living tissues.

### Docking simulation of **NBCeN** into CYP1A1 and CYP1A2

**Fig. 10:**
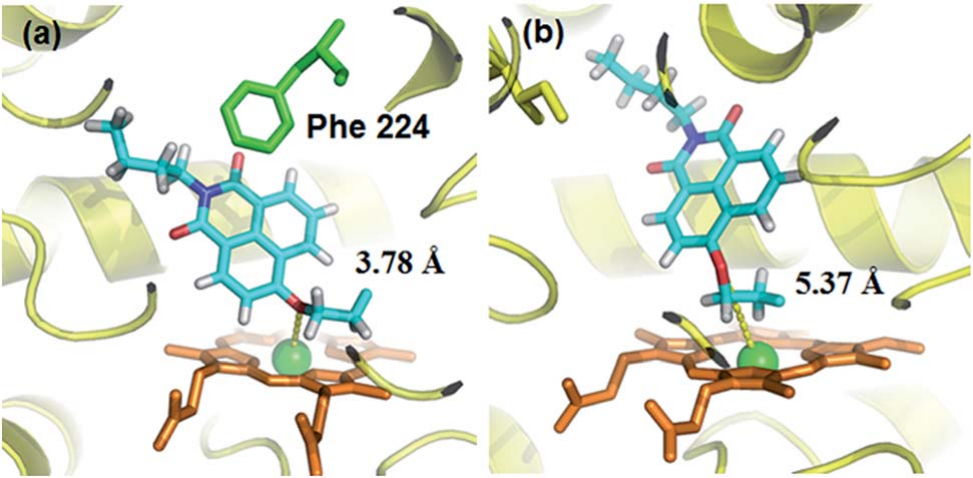
Docking simulation of **NBCeN** into CYP1A1 (a) and CYP1A2 (b). Heme and iron atoms are colored with brown and green, respectively.

A molecular docking simulation was also carried out to explore the difference in **NBCeN**-*O*-dechloroethylation between CYP1A1 and CYP1A2. As shown in Fig. 10, a very complementary π–π stacking interaction was formed between CYP1A1 and **NBCeN** (with the side chain of Phe-224), likely due to an enhanced contact of **NBCeN** with the active site surface of CYP1A1.^26^ Of note, the introduction of chloroethyl groups appeared to orient **NBCeN** markedly better in the active site of CYP1A1 than CYP1A2. Furthermore, the proton-iron distances between the reaction site of **NBCeN** and the heme of CYP1A1 (3.78 Å) were much shorter than those in CYP1A2 (5.37 Å), suggesting that **NBCeN**-*O*-dechloroethylation could occur more readily in CYP1A1. Given that the catalytically reactive distance from the proton-iron of CYP450 is typically within 5.5 Å,^27^ **NBCeN**-*O*-dechloroethylation is hardly likely to occur in CYP1A2. These results are concordant with the experimental results, which displayed that CYP1A1 but not CYP1A2, can catalyze **NBCeN**-*O*-dechloroethylation with a high catalytic efficacy. In addition, the hammerhead score value of the bioactive pose of **NBCeN** in CYP1A1 (9.583) is similar to that in CYP1A2 (8.175), both of which are in agreement with the closed *K*_m_ values for **NBCeN**-*O*-dechloroethylation in both CYP1A1 and CYP1A2. These findings may partially explain why **NBCeN** is a good substrate for CYP1A1 but a poor substrate for CYP1A2.

## Conclusions

In summary, a practical strategy was used to design an isoform-specific two-photon fluorescent probe for highly selective and sensitive detection of CYP1A1 for the first time. The isoform-specific fluorescent probe was designed *via* local modification of the reaction site on a known CYP1A substrate, on the basis of subtle differences in the catalytic cavity and substrate preference between CYP1A1 and other isoforms in the CYP superfamily. Following molecular docking-based virtual screening and reaction phenotyping-based experimental screening of various *O*-alkylated **HN** derivatives, **NBCeN** displayed the best combination of selectivity, sensitivity, high affinity and ratiometric fluorescence response following CYP1A1-catalyzed *O*-dechloroethylation. This newly developed isoform-specific probe could be used to real-time monitor the real activities of CYP1A1 in complex biological systems, and it displayed great potential for high-throughput screening of CYP1A1 modulators using cell or tissue preparations as enzyme sources. Meanwhile, ratiometric TPM bioimaging of **NBCeN** and its metabolite revealed that this probe could serve as an effective imaging tool to monitor endogenous CYP1A1 with high resolution and sensitivity in living cells and tissues. All these findings demonstrated that the newly developed probe could be reliably used for the precise, rapid and highly sensitive detection of CYP1A1 in biomedical and bioanalytical fields, which held a great promise for exploring the biological and physiological roles of endogenous CYP1A1 in living systems. Furthermore, the strategies used in this study are very helpful for the design, screening and optimization of isoform specific probe substrates for target enzyme(s), which opens up a new avenue for the development of isoform-specific probes for other enzymes, especially for hydrolases and lyases.
