# Supplementary material for: A practical strategy to design and develop an isoform-specific fluorescent probe for a target enzyme: CYP1A1 as a case study
Source: Chem Sci. 2016 Dec 19;8(4):2795–803. doi: 10.1039/c6sc03970g (PMC5426458; doi:10.1039/c6sc03970g)
Supplement: Supplementary file 1 [file SC-008-C6SC03970G-s001.pdf]

Electronic Supplementary Material (ESI) for Chemical Science.
This journal is © The Royal Society of Chemistry 2016

Supplementary Information

A practical strategy to design and develop an isoform-specific fluorescent probe for a target enzyme: CYP1A1 as a case study

Zi-Ru Dai, ‡[a,b] Lei Feng, ‡[c] Qiang Jin,[a,b] Hailing Cheng,[d] Yan Li,[a] Jing Ning,[a,b] Yang Yu,[a,b] Guang-Bo Ge,* [a,c] Jing-Nan Cui[c] and Ling Yang*[a]

[a] *Dalian Institute of Chemical Physics, Chinese Academy of Sciences, Dalian, China.*

[b] *Graduate School of Chinese Academy of Sciences, Beijing, China*

[c] *State Key Laboratory of Fine Chemicals, Dalian University of Technology, Dalian, China.*

[d] *Cancer Institute, The Second Hospital of Dalian Medical University, Dalian, China, Dalian, China.*

E-mail: geguangbo@dicp.ac.cn; yling@dicp.ac.cn

Table of Contents

Materials and Chemicals S3

Synthesis and structural characterization of NBCeN. S3

Instruments. S4

Incubation conditions. S5

LC-DAD-ESI-MS analysis. S5

Enzymatic reaction phenotyping assays. S6

Chemical inhibition assays S6

Determination of the limit of detection. S7

Enzyme kinetics analysis. S7

Correlation studies. S7

Cytotoxicity assays S8

Cell culture and confocal fluorescence imaging S8

Tissue slices preparation and two-photon fluorescence imaging. S9

Molecular docking S9

Figures (Fig.S1-S17). S10-19

Tables S20-22

References S22

Structure characterization S23-24

Materials and methods

Materials and chemicals. All chemicals were commercial products of analytical grade. cDNA-expressed recombinant human CYP1A1, CYP1A2, CYP1B1, CYP2A6, CYP2A13, CYP2B6, CYP2C8, CYP2C9, CYP2C19, CYP2D6, CYP2E1, CYP2J2, CYP3A4, CYP3A5, CYP4F2, CYP4F3 and CYPex derived from baculovirus-infected insect cells co-expressing NADPH-CYP reductase and cytochrome b5 were obtained from BD Biosciences (Woburn, MA, USA). Pooled human lung microsomes prepared from mixed gender (10 donors) were obtained from Bioreclamation IVT (Baltimore, MD, USA). Pooled human liver microsomes prepared from mixed gender (50 donors), a panel of twelve HLMs from individuals (male Mongulia) and pooled rat microsomes (RLM) were obtained from Research Institute for Liver Diseases (RILD, Shanghai, China). 1-Aminobenzotriazole (ABT), sulfaphenazole, quinidine, furafylline, resveratrol, α-Naphthoflavone, tranylcypromine, omeprazole, glucose-6-phosphate dehydrogenase, NADP+, D-glucose-6-phosphate and trypsin (TPCK-treated, from bovine pancreas) were purchased from Sigma (St. Louis, MO, USA). Clomethiazole was obtained from ICN Biomedicals Inc (Aurora, OH, USA). Montelukast was from Beijing Aleznova Pharmaceutical (Beijing, China). Triethylenethiophosphoramide (TEPA) was purchased from Acros Organics (Geel, Belgium). 7-ethoxyresorufin (EROD) was purchased from Toronto Research Chemicals (Toronto, ON.CAN). 2,3,7,8-tetrachlorodibenzo-p-dioxin (TCDD) was purchased from AccuStandard (New haven, CT, USA). A549, HepG2 and SKOV cells were purchased from the Committee on Type Culture Collection of Chinese Academy of Sciences (Shanghai, China). All other reagents, fine chemicals and LC solvents with the highest grade commercially available were obtained from J&K Chemical Ltd (Shanghai, China) and Tedia (Fairfield, OH, USA).

Synthesis and structural characterization of NBCeN. A mixture of N-(4-butyl)-4- hydroxy -1, 8-naphthalimide (135 mg, 0.5 mmol), 1-chloro-2-iodoethane (476 mg,2.5 mmol) and K_2CO_3 (691 mg, 3 mmol) in 10 mL CH_3CN was refluxed overnight. After cooling to room temperature, the solvent was removed in vacuo, and the residual solid was purified by chromatography (silica gel, EtOAc–

hexane as eluent, 1:5, v/v) to afford **NBCeN** as a yellow solid. The structures of **NBCeN** were confirmed by ^{1}H NMR, ^{13}C NMR and HRMS spectroscopy, and the data are as follows: ^{1}H NMR (400 MHz, $CDCl_3$) δ 8.59 (s, 2H), 8.51 (d, J = 7.9 Hz, 1H), 7.71 (t, J = 7.6 Hz, 1H), 7.00 (d, J = 8.1 Hz, 1H), 4.53 (t, J = 5.2 Hz, 2H), 4.16 (t, J = 7.4 Hz, 2H), 4.02 (t, J = 5.4 Hz, 2H), 1.71 (dt, J = 15.0, 7.6 Hz, 2H), 1.59 – 1.38 (m, 2H), 0.98 (t, J = 7.3 Hz, 3H). ^{13}C NMR(100 MHz,$CDCl_3$) δ164.41, 163.82, 159.12, 133.05, 131.66, 129.37, 128.53, 126.19, 123.40, 122.47, 115.80, 105.90, 68.64, 41.60, 40.14, 30.27, 20.42, 13.89. HRMS calcd for $C_{18}H_{19}ClNO_3^+$ ($[M+H]^+$) 332.1048, found 332.1041.

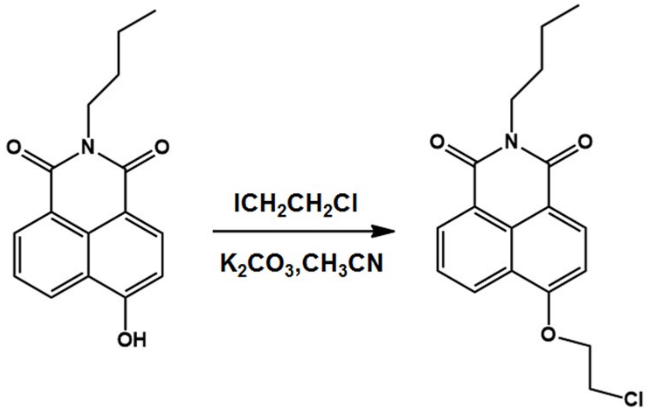

Scheme S1 The synthesis procedure of **NBCeN**.

Instruments. ^{1}H-NMR and ^{13}C-NMR spectra were recorded using Bruker Avance II spectrometer (400 MHz for ^{1}H NMR and 100 MHz for ^{13}C NMR) spectrometer with chemical shifts reported as ppm (in $CDCl_3$; TMS as internal standard). High-resolution mass spectral (HR-MS) analyses were measured with Hybrid Ion Trap-Orbitrap Mass Spectrometer (LTQ Orbitrap XL, Thermo). LC-DAD-ESI-MS measurements were performed on a Shimadzu UFLC system coupled with a diode array detector and a mass spectrometer (Shimadzu 2010 EV, Kyoto, Japan). Absorption spectra and fluorescence emission/excitation spectra were measured on BioTek Synergy H1 Hybrid Multi-Mode Microplate Reader. The fluorescence images of cells were taken using a FV1000 MPF confocal laser scanning microscopy (Olympus, Tokyo, Japan) with an objective lens (×60). Stock solution of **NBCeN** (10 mM) was prepared in acetonitrile and stored at -80 °C for future using. All microsomal samples and recombinant human CYP isoforms were prepared in phosphate buffer (pH

7.4) and stored at -80 °C.

Incubation conditions. The incubation mixture, with a total volume of 200 µl, consisted of 100 mM potassium phosphate buffer (pH 7.4), NADPH-generating system (1 mM $NADP^+$, 10 mM glucose-6-phosphate, 1 unit/mL of glucose-6-phosphate dehydrogenase, and 4 mM $MgCl_2$), and human tissue microsomes or CYPs. In all experiments, **NBCeN** (20 mM dissolved in acetonitrile previously) was serially diluted to the required concentrations and the final concentration of acetonitrile did not exceed 1% (v/v) in the mixture. After preincubation at 37ºC for 3 min, the reaction was initiated by adding NADPH-generating system and further incubated at 37ºC in a shaking water bath. The reaction was terminated by the addition of ice-cold acetonitrile (100 µl). The mixture was kept on ice until it was centrifuged at 20,000×g for 20 min at 4ºC. Aliquots of supernatants were stored at -20ºC until analysis. Aliquots of supernatants were taken for further fluorescence analysis. Control incubations without NADPH-generating system or without substrate or without CYP enzyme sources were carried out to investigate whether the formation of metabolite(s) was enzyme (CYP)- and NADPH-dependent. All incubations throughout the study were carried out in duplicate with SD. values generally below 10%.

LC-DAD-ESI-MS analysis. **NBCeN** and its dechloroethylation metabolite in CYP1A1 were identified by using LC-DAD-ESI-MS. The incubation conditions and procedures were used as mentioned above. The supernatants were analyzed using a Shimadzu UFLC system coupled with diode array detector and mass spectrometer. A Shim-pack XR-ODS (2 µm, 2.0 ×150.0 mm; Shimadzu, Kyoto, Japan) analytical column was used and the column temperature was kept at 40 °C. The mobile phase consisted of CH_3CN (A) and water containing 0.2% formic acid (B) using a flow rate at 0.4 mL/min. **NBCeN** and its dechloroethylation metabolite can be quantified by using this LC-UV method monitored at 243 nm. The mass spectrometer was operated under the positive ion monitoring mode from *m/z* 50 to 1000. The detector voltage was set at +1.55 kV for positive ion detection. The curved desolvation line temperature (CDL) and the block heater temperature

were both set at 250 °C. Other MS detection conditions were as follows: interface voltage, 4 kV; CDL voltage, 40 V; nebulizing gas (N_2) flow was 1.5 L/min and the drying gas (N_2) pressure was set at 0.06 MPa. Data processing was performed using the software LC-MS Solution version 3.41 (Shimadzu, Kyoto, Japan).

Enzymatic reaction phenotyping assays. Seventeen cDNA-expressed human CYP isoforms co-expressing NADPH-P450 reductase (CYP1A1, CYP1A2, CYP1B1, CYP2A6, CYP2A13, CYP2B6, CYP2C8, CYP2C9, CYP2C19, CYP2D6, CYP2E1, CYP2J2, CYP3A4, CYP3A5, CYP4F2, CYP4F3 and CYPex) were used to screen the involved isoform(s) for dechloroethylation of **NBCeN** in human tissue microsomes. The incubations were carried out under the above mentioned incubation conditions with each of CYP isoform. To generate adequate metabolites for detection, a relative high substrate concentration (20 μM) was used and incubated with each of the recombinant CYPs (40-80 nM) at 37ºC for 30 min.

Chemical inhibition assays. O-dechloroethylation of **NBCeN** in pooled HLM in the absence or presences of selective inhibitors for different CYP isoforms were measured to verify the involved enzyme(s) for this biotransformation. In brief, **NBCeN** (20 μM) was incubated in HLM (0.25 mg/mL) with an NADPH-generating system in the absence (control) or presence of known CYP isoform-specific inhibitors. The selective inhibitors and their concentrations were as follows: resveratrol (20 μM) for CYP1A1, montelukast (2 μM) for CYP2C8, sulfaphenazole (10 μM) for CYP2C9, omeprazole (20 μM) for CYP2C19, quinidine (10 μM) for CYP2D6 and clomethiazole (50 μM) for CYP2E1. Inhibition by furafylline (10 μM) for CYP1A2, tranyleypromine (1 μM) for CYP2A6,[1] TEPA (50 μM) for CYP2B6, troleandomycin (200 μM) for CYP3A and ABT (500 μM) for broad CYPs were assayed by pre-incubation with NADPH-generating system at 37ºC for 20 min. Other procedures including termination step and sample preparation were depicted as described previously. The inhibitory effects were expressed as percent decrease in fluorescence intensities ratio ($\boldsymbol{I}_{564}/\boldsymbol{I}_{452}$). Data were fit to log (inhibitor) vs. normalized response -- Variable slope

equation using GraphPad Prism 6 (GraphPad Software, San Diego, CA).

Determination of the limit of detection. The assay was performed on a plastic 96-well microplate (Costar, half area black plate). A mixture of **NBCeN**, NADPH-generating system, recombinant CYP1A1 (2.5 nM to 50 nM) in potassium phosphate buffer (100mM, pH 7.4) containing less than 1% acetonitrile (total volume 200 μL) was incubated for 60 min at 37 °C. Fluorescence measurement was performed at the end of the incubation, using a BioTek Synergy H1 Hybrid Multi-Mode Microplate Reader, and the average fluorescence intensity of each group was compared with that of the control group without CYP1A1.

Enzymatic kinetic analysis. To estimate the kinetic parameters of **NBCeN**-O-dechloroethylation in different enzyme sources, the incubation conditions were optimized to ensure the formation rates of **NBHN** was in the linear range in relation to incubation time and protein concentration at 37 °C. **NBCeN** (dissolved in acetonitrile previously) was serially diluted to the required concentrations (0.1, 0.25, 0.5, 1.0, 2.5, 5, 10, 25 μM), and the final concentration of acetonitrile was 1% (v/v). **NBCeN** was incubated with pooled human liver microsomes and pooled human lung microsomes (0.25 mg protein/mL) for 30 min, or with recombinant CYP1A1 (75 nM) for 30 min. All incubations were performed in duplicate, and the fluorescence intensity was recorded continuously as described above. The relationship between [S] and [V] was plotted, and fitted to the Michaelis-Menten equation. Data analysis was performed by a nonlinear regression using GraphPad Prism 6 (GraphPad Software, San Diego, CA).

$$v = \frac{\mathrm{V}_{\mathrm{max}} \times [S]}{K_{\mathrm{m}} + [S]}$$

Correlation studies. In order to evaluate the applicability of **NBCeN** as a selective indicator for measurement of CYP1A1 activity in human biological samples, the formation rates of the metabolite(s) described for **NBCeN** in a panel of twelve HLMs from individuals were determined

and compared with the levels of CYP1A1. The dechloroethylation of **NBCeN** (10 μM) was performed in HLMs (0.25 mg/mL) for 30 min. The concentrations of CYP1A1 in HLM were determined by liquid chromatography-tandem mass spectrometer (LC-MS/MS), using multiple reaction monitoring (MRM) mode and isotope labeled peptide as the internal standards. Specific peptides of YLPNPSLNAFK (for CYP1A1) were selected for their quantification by using transition ion of 632.5/776.4. Then the rates of **NBCeN** dechloroethylation in 12 individual HLMs were compared with the levels of CYP1A1. The correlation parameter was expressed by the linear regression coefficient (R^2). $P < 0.0001$ was considered statistically significant.

Cytotoxicity assays. Cell viability was investigated by 3-(4,5)-dimethylthiahiazo (-z-y1)-3,5-di-phenytetrazoliumromide (MTT) assay.[2] A549 (human lung cancer line) and SKOV (human adenocarcinoma cell line) cells were seeded in a 96-well plate at a concentration of 5×10^4/mL in 200 μL of Dulbecco's modification of Eagle's medium Dulbecco (DMED) medium with 10% FBS and maintained at 37 °C in a 5% CO_2 incubator for 24 h. Then the A549 and SKOV cells were incubated with different concentrations of **NBCeN** (0, 0.01, 0.1, 1, 10, 20, 50, 100 μM) for 48 h. The cells were washed once with 37 °C PBS and then added 0.1 mL serum-free medium containing 0.05% MTT to each well. After incubation for 4 h, the culture medium was removed and 0.15 mL of DMSO was added to each well to solubilize the formazan formed. The plates were shaken gently for 10 min and the absorbance was measured at 490 nm. The absorbance of treated cells was compared with the absorbance of the controls, which cells were exposed only to the vehicle and were considered as 100% viability value.

Cell culture and confocal fluorescence imaging. A549 and SKOV were grown in Dulbecco's modification of Eagle's medium Dulbecco (DMEM/high: with 4.5 g/L Glucose, 4.0 mM L-Glutamine, and 110 mg/L Sodium Pyruvate), supplemented with fetal bovine serum of 10%. Washed twice with FBS-free DMEM, the adherent cells were incubated with/without 50 μM resveratrol (preparing in FBS-free DMEM) for 60 min at 37 °C in CO_2 incubator of 5%. At a 50

μM final concentration, stock solution of probe **NBCeN** (20 mM) in DMSO was diluted into the cell culture media (FBS free). The cells were then incubated at 37 °C for another 60 min, and then washed with PBS (pH 7.4) for three times, and then imaged on confocal microscope (Olympus, FV1000, Japan). In one photon microscopy assay, excitation wavelength was set at 405 nm, blue emission was collected with a 420-470 nm window, and green emission was collected with a 530-580 nm window. In two-photon mode, images were acquired under 770 nm excitation and fluorescent emission windows of 420-460 nm (blue) and 495-540 nm (green).

Tissue slices preparation and two-photon fluorescence imaging. Slices were prepared from the liver of 7-weeks-old rat. Slices were cut to 100 μm thickness by a vibrating-blade microtome in 25 mM PBS (pH 7.4). For the control experiments, slices were incubated with 20 mM **NBCeN** in PBS buffer bubbled with 95% O_2 and 5% CO_2 at 37 °C for 1h. Slices were then washed three times with PBS, transferred to glass-bottomed dishes (Nest, 35 mm dish with 20 mm well), and observed under a two-photon confocal microscope (Olympus, FV1000, Japan).

Molecular docking. Docking simulation was performed by using SYBYL (X-1.1). Here, the X-ray crystallographic structure of human cytochrome P450 1A1 (coded as 4I8V) and P450 1A2 (coded as 2HI4) were selected for the docking study. After the backbone and variable modeling, a 1000-step minimization was carried out to obtain a low-energy conformation without any steric clashes between side chains. With the crystal structure of 1A1 and 1A2, the bioactive binding conformations of (1), (2), (3), (4), (5) and (6, **NBCeN**) were generated using Surflex-Dock, which were evaluated by proton-iron distance, one of the most suitable scoring functions for P450s super-family.

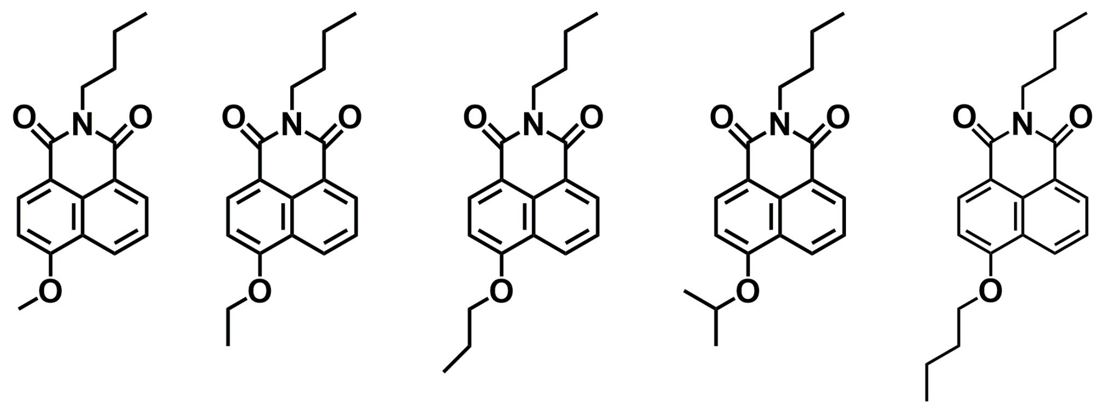

1 **2** **3** **4** **5**

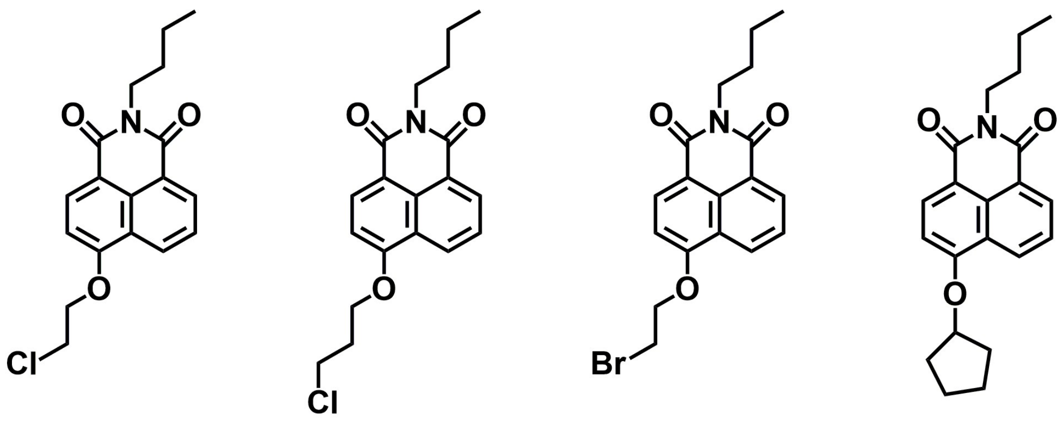

6 **7** **8** **9**

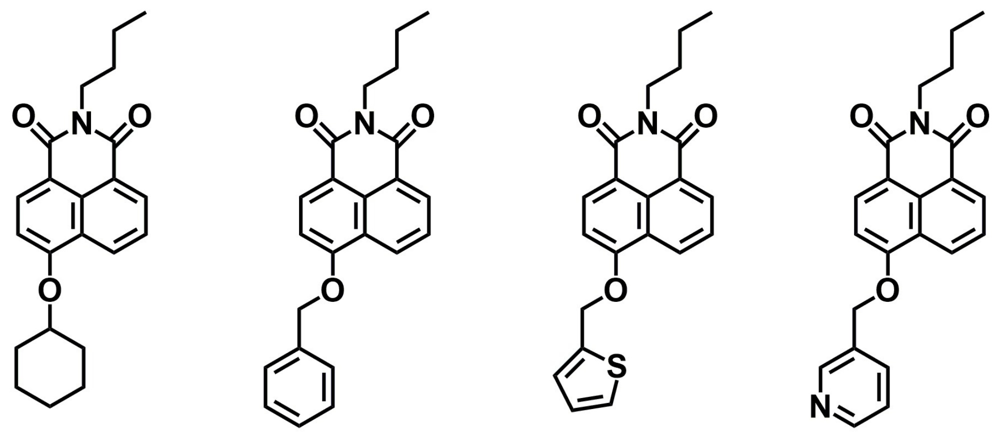

10 **11** **12** **13**

Fig. S1 Chemical structures of 4-hydroxy-1,8-naphthalimide derivatives used in this study.

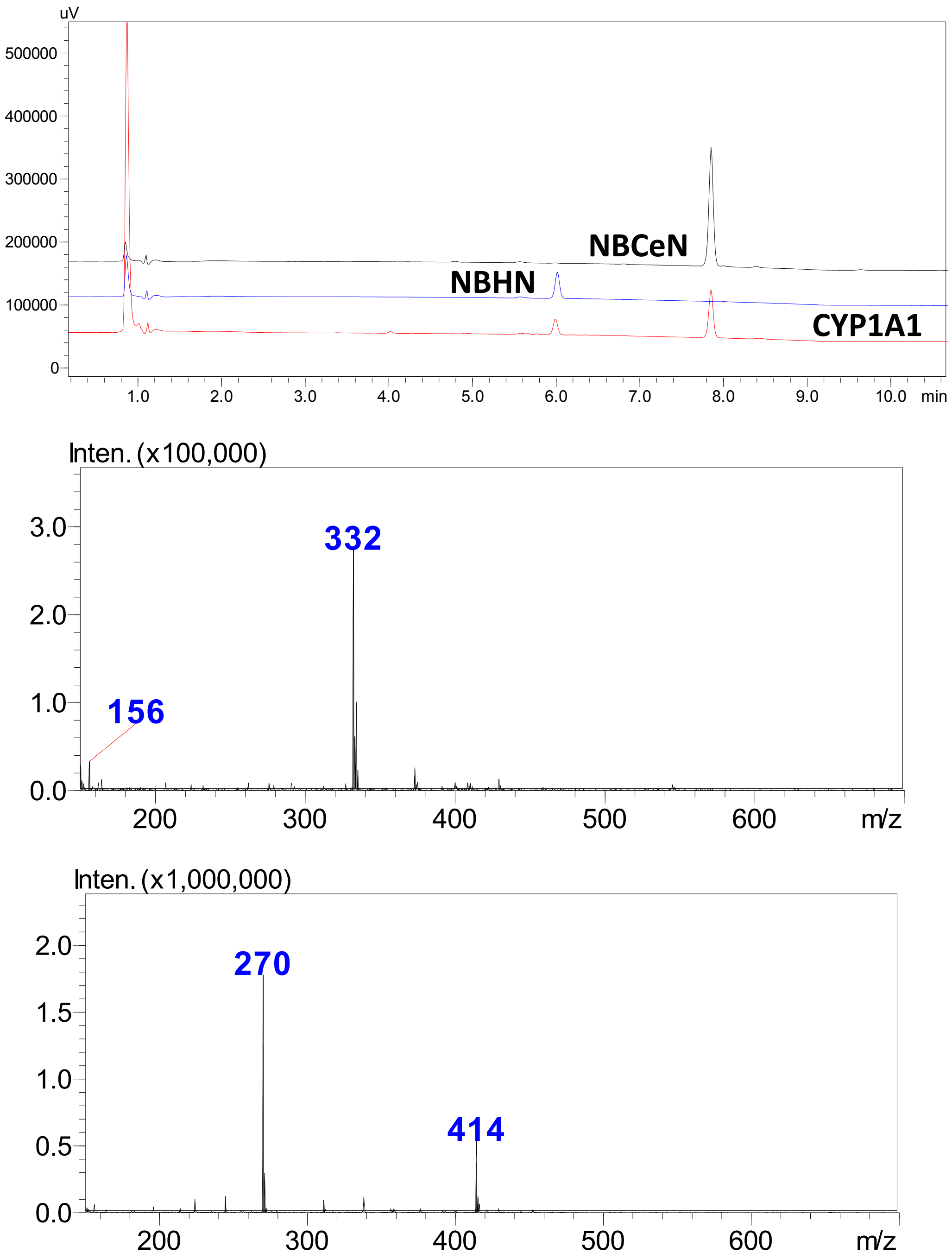

Fig. S2 LC-ESI-MS analysis of the reaction mixtures of **NBCeN**. LC chromatograms of the incubation samples (1. **NBCeN** standard, 2. **NBHN** standard, 3. **NBCeN** (20 μM) incubation in CYP1A1 100nM) for 30 min, The detection wavelength was set at 243 nm. The mass spectra of **NBCeN** and its metabolite were recorded under positive ion mode.

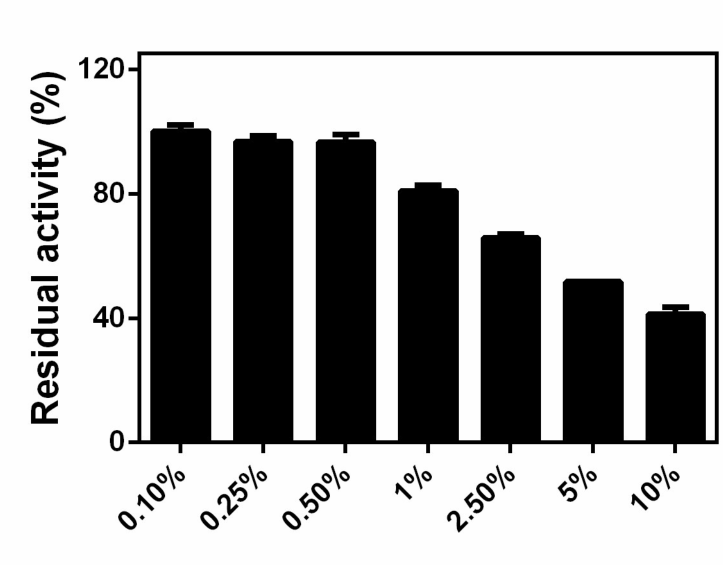

Fig S3. The effects of DMSO concentration on the hydrolytic rates of **NBCeN** (20 μM) upon addition of CYP1A1.

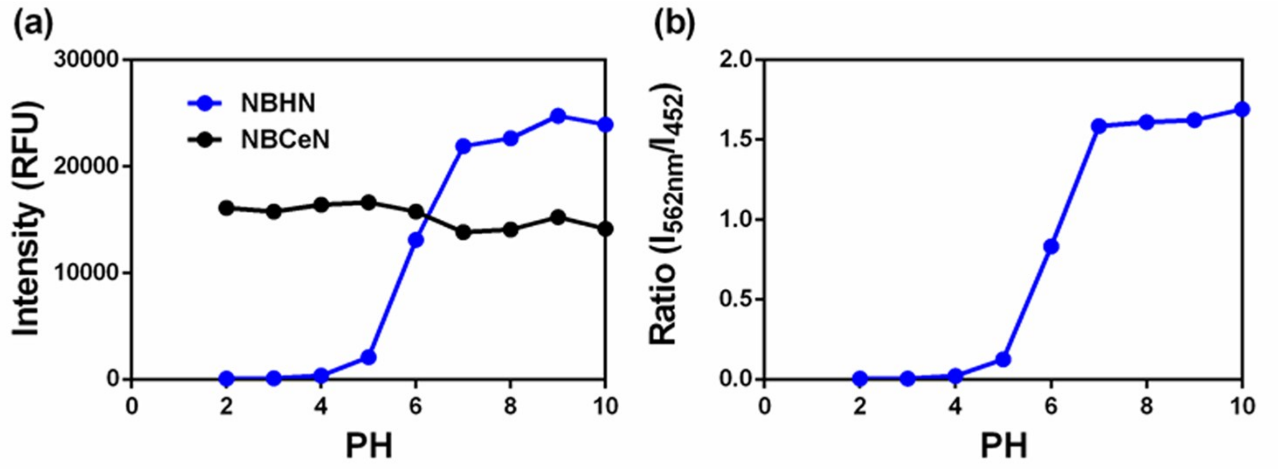

Fig. S4. Effects of pH on the fluorescence intensities ratio of **NBCeN** (20 μM) and its metabolite **NBHN** (20 μM). The measurements were performed in KCl-HCl buffer- acetonitrile (v/v=1:1) with different pH values adjusted by HCl and KOH. Excitation wavelengths for **NBCeN** and its metabolite were 372 nm (Gain = 80) and 450 nm (Gain = 100), respectively.

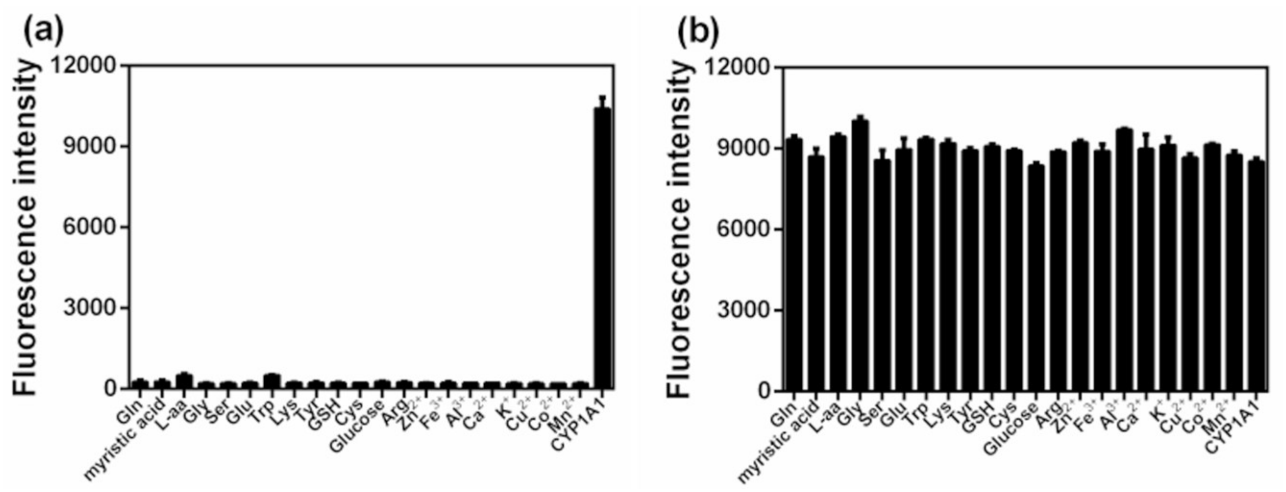

Fig S5. Fluorescence responses of **NBCeN** (20 μM) to various analytes in aqueous solution (acetonitrile:PBS = 1:1) (a). Fluorescence responses of NBCeN (20 μM) towards CYP1A1 in the presence of various analytes in aqueous solution (acetonitrile:PBS = 1:1) (b).

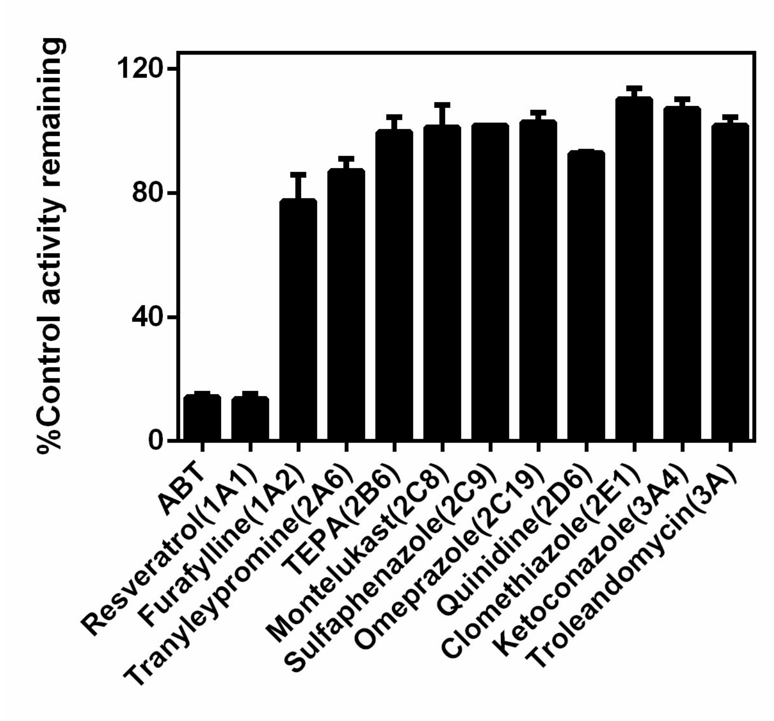

Fig S6. Inhibitory effects of selective CYP inhibitors on **NBCeN** (20 μM) O- dechloroethylation HLM (0.25 mg/mL). The spectra were measured in PBS-acetonitrile (v: v = 1: 1, pH 7.4) at 37 °C for 60 min. λ_{ex} = 372/452 nm.

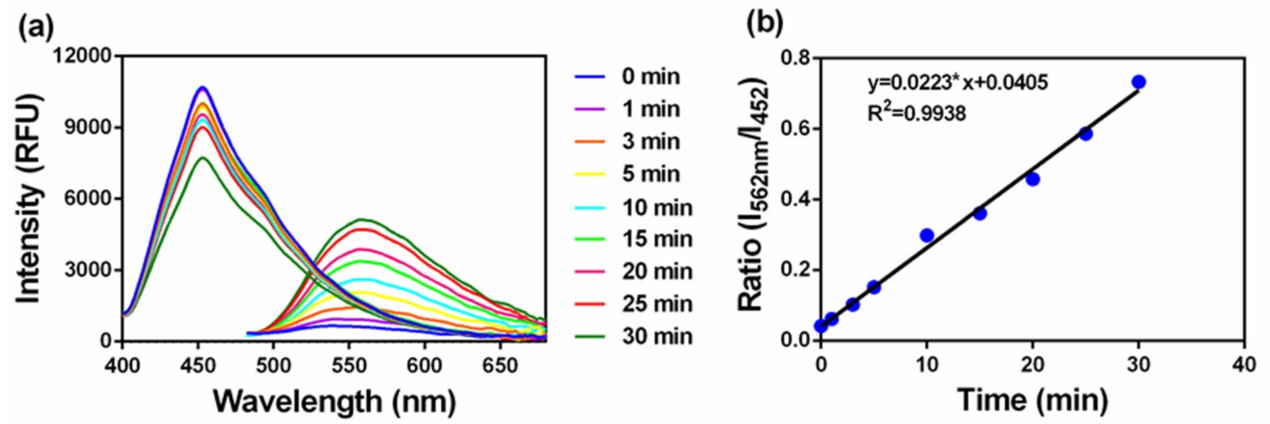

Fig. S7. Fluorescence spectra changes of **NBCeN** (10 μM) against the reaction time in CYP1A1 (a). Time-dependent fluorescence intensity ratios change at 452 nm and 562 nm ($\boldsymbol{I}_{562\text{ nm}}/\boldsymbol{I}_{452\text{ nm}}$) of **NBCeN** to **NBHN** (10 μM) in CYP1A1 (b).

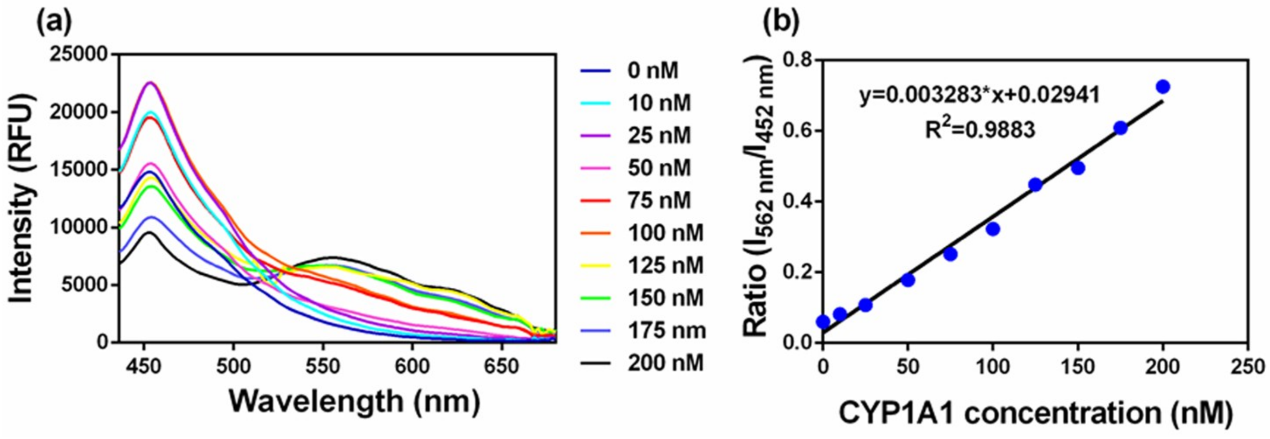

Fig **S8**. (a) The changes in fluorescence spectra and (b) fluorescence intensity ratios ($\boldsymbol{I}_{562\text{ nm}}/\boldsymbol{I}_{452\text{ nm}}$) of **NBCeN** (20 μM) upon addition of increasing concentrations of CYP1A1 (0 -200 nM) in PBS–acetonitrile (v: v = 1: 1, pH 7.4) at 37 °C. λ_{ex} = 405 nm.

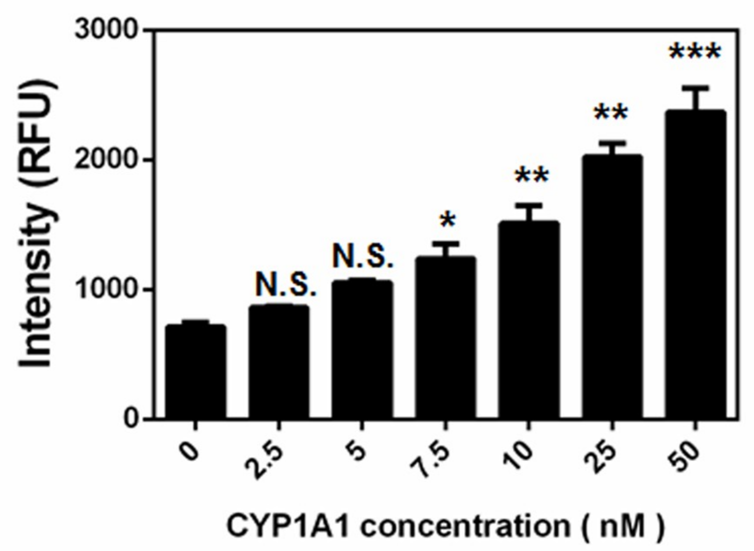

Fig. S9. Detection limit of CYP1A1 (a) with **NBCeN** as the substrate on a 96-well plate. Data are shown as mean ± S. D. (n = 5). ** indicates $p < 0.005$ and *** indicates $p < 0.001$, versus the control group without enzyme (one-sided Student's t-test). λ_{ex} = 405 nm.

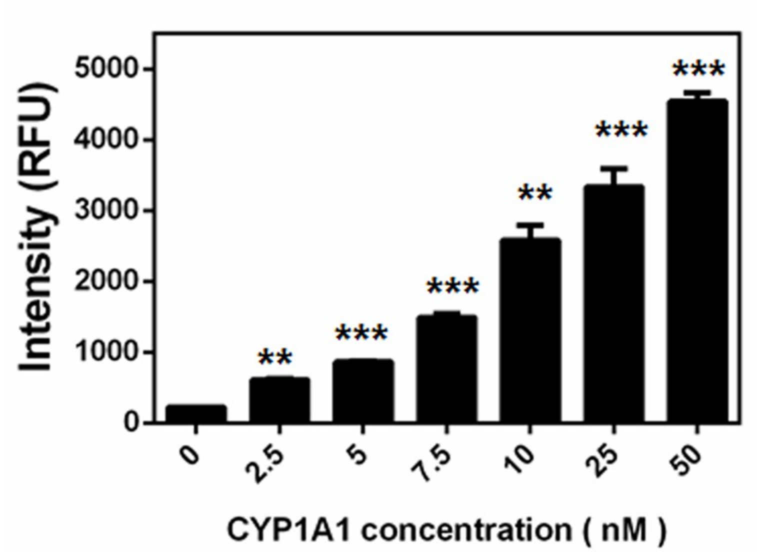

Fig. S10. Detection limit of CYP1A1 (a) with **NBCeN** as the substrate on a 96-well plate. Data are shown as mean ± S. D. (n = 5). ** indicates $p < 0.005$ and *** indicates $p < 0.001$, versus the control group without enzyme (one-sided Student's t-test). λ_{ex} = 372/452 nm.

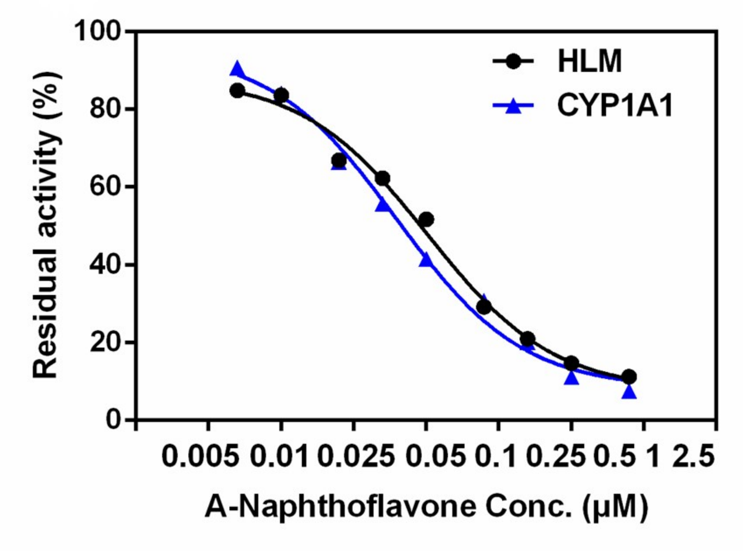

Fig. S11. Dose-inhibition curves of α-naphthoflavone (0.005-2.5 μM) on **NBCeN**-O-dechloroethylation in both HLM (0.25 mg/mL) and CYP1A1 (100 nM).

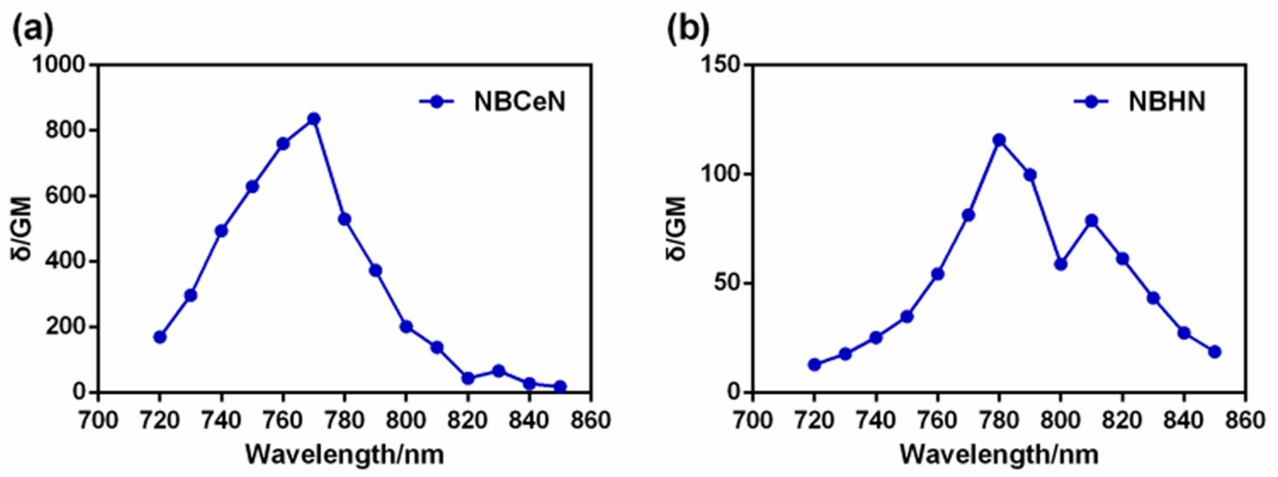

Fig. S12 Two-photon action spectra of **NBCeN** (a) and **NBHN** (b) in acetonitrile/ PBS (5/5, v/v, 100 mM, pH 7.4).

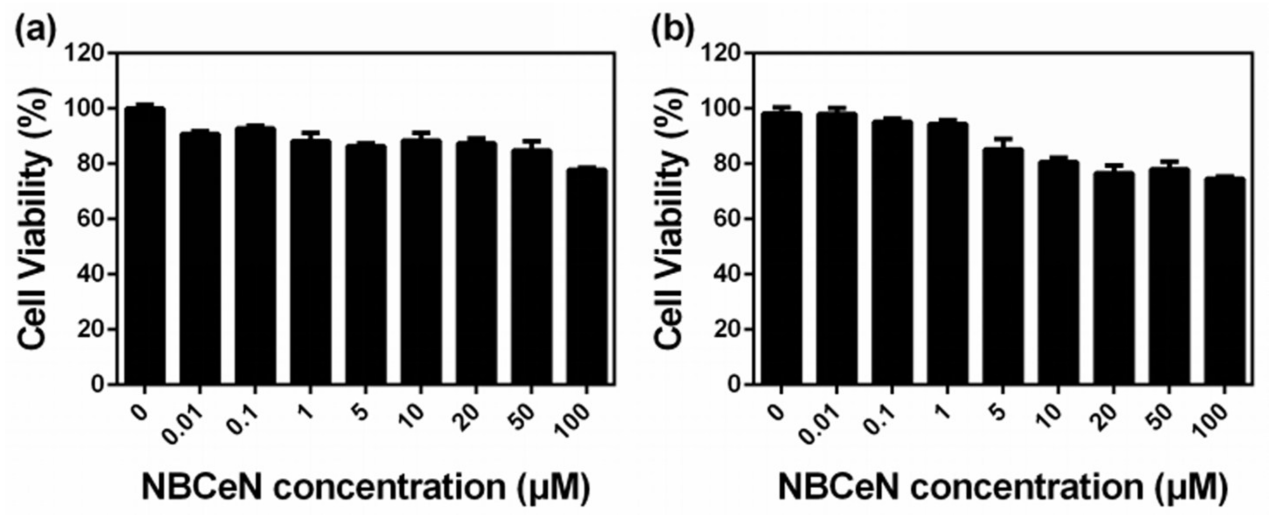

Fig. S13. Cell viability of A549 cells and SKOV cells in the presence of different concentrations of **NBCeN** (a) and (b) were determined by using a standard MTT assay.

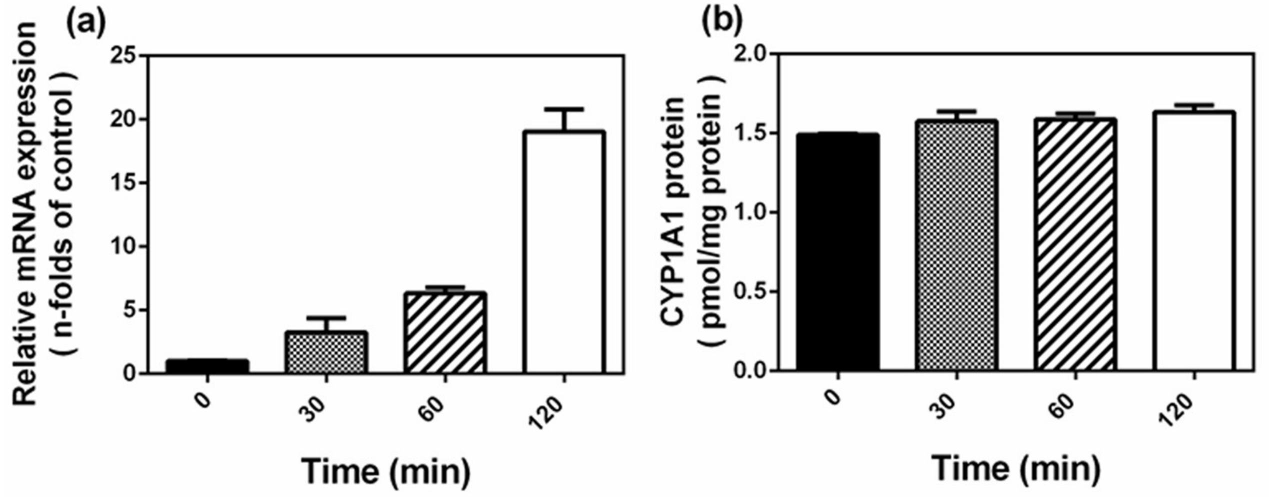

Fig. S14. (a) The mRNA levels of CYP1A1 in A549 cells following pre-treatment with **NBCeN** (50 μM) or vehicle solvent (DMSO) at different time points (0, 30 min, 60 min and 120 min). (b) The levels of CYP1A1 in A549 cells were determined by an ELISA-based method. Data are shown as mean ±S.D. (n=3).

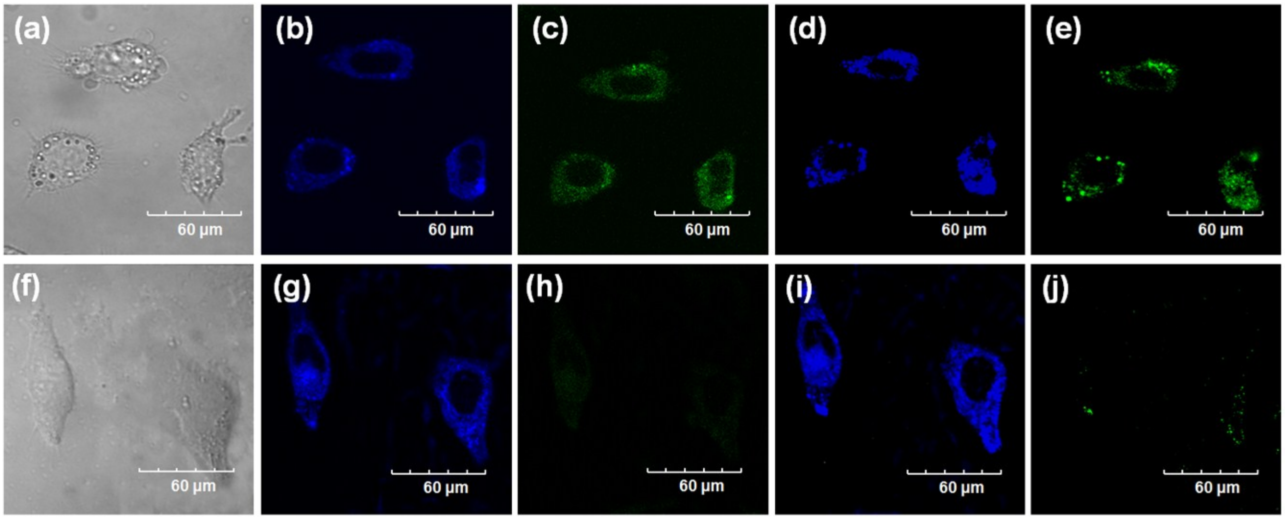

Fig S15. Confocal fluorescence images of A549 cells. Cells incubated with **NBCeN** (50 µM) for 1h (a-e); cells pre-treated with resveratrol (5 µM) (f-j) for 1h and then incubated with **NBCeN** for 1h, respectively. Single photon Images were acquired using 405 nm excitation and fluorescent emission windows: (a) and (f) bright-field images; (b) and (g) blue emission channel; (c) and (h) green emission channel. Two photon images were acquired using 770 nm excitation and fluorescent emission windows: (d) and (i) blue= 420–460 nm; (e) and (j) green= 495–540 nm. Scale bar: 60 µm.

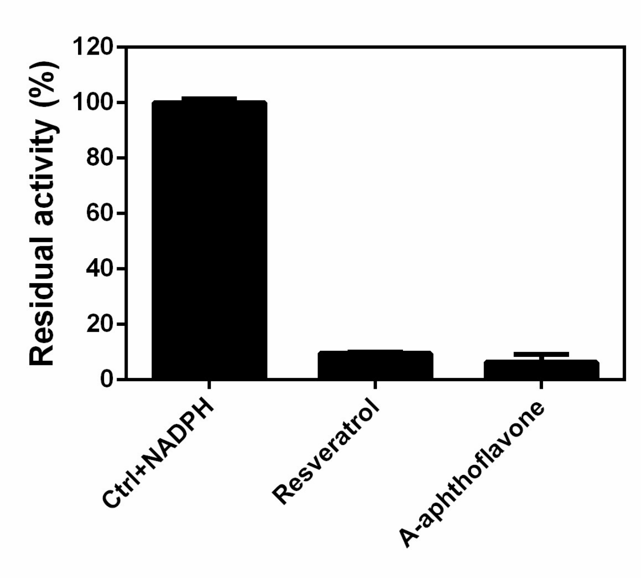

Fig. S16. Inhibitory effects of CYP1A1 inhibitors (50 μM) on **NBCeN** (50 μM) O-dechloroethylation in RLM. Data represent the mean of duplicate incubations. The spectra were measured in PBS-acetonitrile (v/v=1:1, pH 7.4) at 37 °C for 30 min.

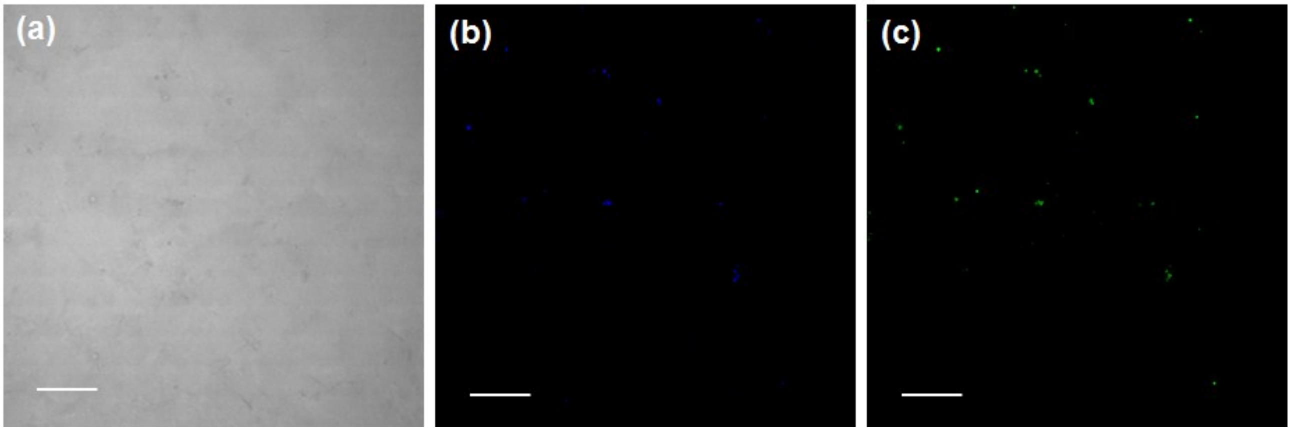

Fig. S17. TPM images of a fresh rat liver slice. All images were taken with magnification at 60×. Two photon Images were acquired following excitation at 770 nm and the fluorescent emission windows as follows: bright-field images (a); blue= 420–460 nm (b); green= 495–540 nm (c). Scale bar: 30 μm.

Table S1 4-hydroxy-1,8-naphthalimide derivatives related parameters derived from the molecular modeling of 4-hydroxy-1,8-naphthalimide derivatives with the crystal complex of CYP1A1, respectively.

Compound	Site-heme distance	Hammerhead score	π-π stacking interaction
**1**	**3.48 Å**	**7.3162**	**Phe 224**
**2**	**4.66 Å**	**9.3236**	**Phe 224**
**3**	**5.23 Å**	**9.3058**	**Phe 224**
**4**	**5.37 Å**	**9.3309**	**Phe 224**
**5**	**5.37 Å**	**9.5044**	**Phe 224**
**6**	**3.78 Å**	**9.5832**	**Phe 224**
**7**	**4.49 Å**	**8.0692**	**Phe 224**
**8**	**3.97 Å**	**9.5832**	**Phe 224**
**9**	**6.74 Å**	**7.5385**	**Phe 224**
**10**	**6.95 Å**	**11.1307**	**Phe 224**
**11**	**5.61 Å**	**10.5714**	**Phe 224**
**12**	**6.82 Å**	**9.9639**	**Phe 224**
**13**	**6.03 Å**	**9.9564**	**Phe 224**

Table S2 4-hydroxy-1,8-naphthalimide derivatives related parameters derived from the molecular modeling of 4-hydroxy-1,8-naphthalimide derivatives with the crystal complex of CYP1A2, respectively.

Compound	Site-heme distance	Hammerhead score	π-π stacking interaction
**1**	**4.62 Å**	**8.0912**	
**2**	**5.12 Å**	**8.6945**	**Phe 226**
**3**	**6.08 Å**	**8.6712**	**Phe 226**
**4**	**6.26 Å**	**8.8105**	**Phe 226**
**5**	**6.25 Å**	**10.2530**	**Phe 226**
**6**	**5.37 Å**	**8.1748**	
**7**	**5.27 Å**	**7.7507**	**Phe 226**
**8**	**5.60 Å**	**9.2087**	**Phe 226**
**9**	**7.00 Å**	**9.8537**	**Phe 226**
**10**	**7.07 Å**	**4.8050**	**Phe 226**
**11**	**7.35 Å**	**10.5666**	**Phe 226**
**12**	**10.36 Å**	**3.9641**	**Phe 226**
**13**	**7.22 Å**	**10.0779**	**Phe 226**

Table S3 Isoform specificity of 4-hydroxy-1,8-naphthalimide derivatives used in this study.

Enzyme \ Compound	1	2	3	4	5	6	7	8
CYP1A1	+++	+++	+++	+++	+++	+++	+++	++
1A2	++	++	++	+	++	-	-	-
1B1	++	+	-	-	-	-	+	-
2A6	-	-	-	-	+	-	-	+
2A13	-	-	-	-	-	-	-	-
2B6	+++	+	++	++	-	-	+	-
2C8	-	-	-	-	-	-	-	-
2C9	-	-	-	-	-	-	+	-
2C19	++	++	+	+	-	-	++	-
2D6	++	+	+	+	+	-	+	+
2E1	-	-	-	-	-	-	-	-
2J2	-	-	-	-	-	-	++	-
3A4	-	+	++	++	-	-	-	-
3A5	-	-	-	-	-	-	-	-
4F2	-	-	-	-	+	-	-	-
4F3	-	-	-	-	-	-	-	-

Fluorescence intensity: -:＜200; +: 200-500; ++: 500-2000; +++:＞2000.

Table S4 LOD of CYP1A1 by using different methods

**Quantification method**	**LOD**
Excited at two different wavelengths (λ_{ex} = 372/452 nm)	**2.5 nM**
Excited at one wavelength (λ_{ex} = 405 nm)	**7.5 nM**

References

(1) Zhang, W. J.; Kilicarslan, T.; Tyndale, R. F.; Sellers, E. M. *Drug Metabolism and Disposition*.**2001**, *29*, 897.

(2) Fan, J. L.; Zhan, P.; Hu, M. M.; Sun, W.; Tang, J. Z.; Wang, J. Y.; Sun, S. G.; Song, F. L.; Peng, X. J. *Org Lett*.**2013**, *15*, 492.

Structural characterization

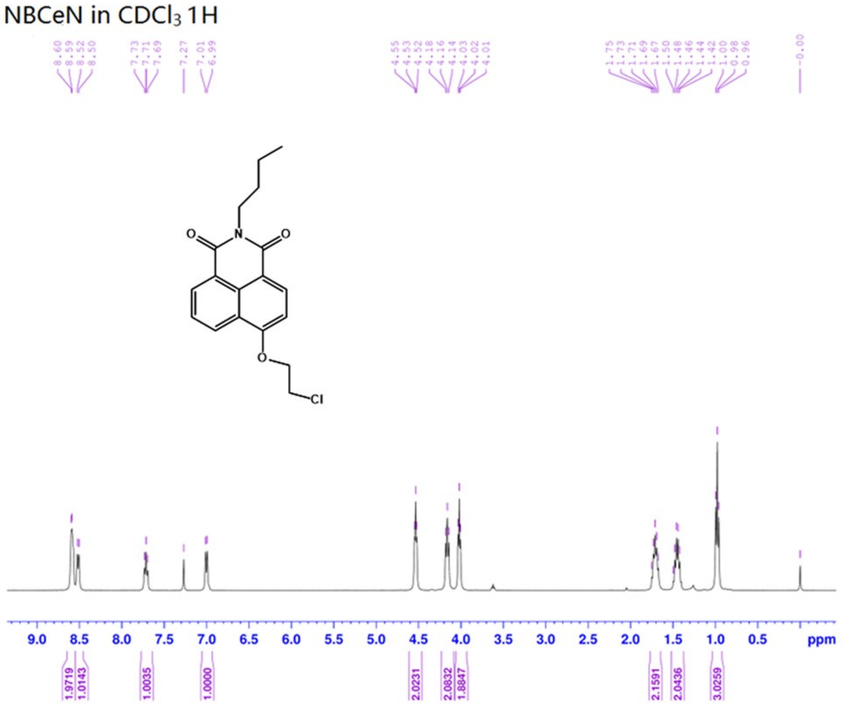

^{1}H-NMR (400 MHz, $CDCl_3$) spectrum of **NBCeN**.

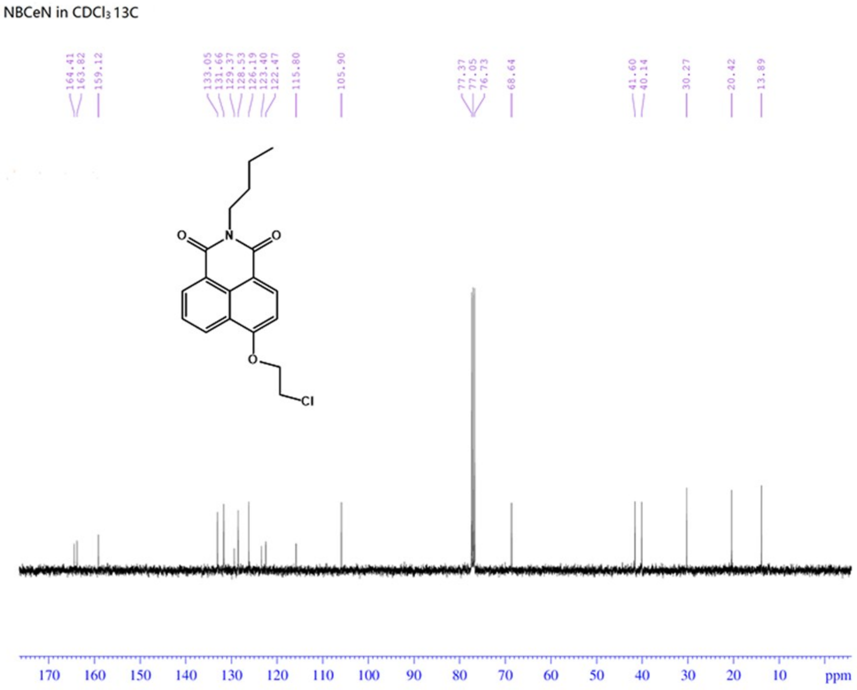

^{13}C-NMR (100 MHz, $CDCl_3$) spectrum of **NBCeN**.

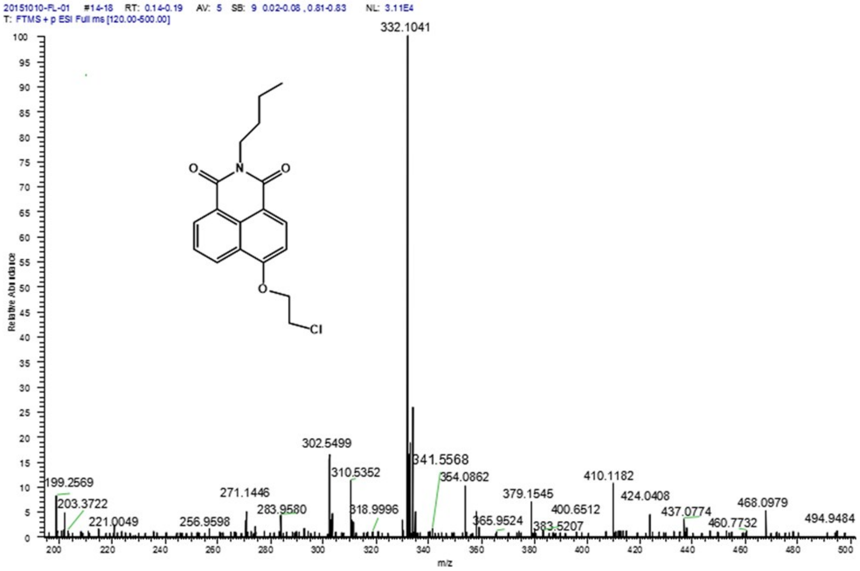

HRMS spectrum of **NBCeN**.
